# Identification and synthesis of impurities formed during sertindole preparation

**DOI:** 10.3762/bjoc.7.5

**Published:** 2011-01-07

**Authors:** I V Sunil Kumar, G S R Anjaneyulu, V Hima Bindu

**Affiliations:** 1 Research and Development Centre, Aptuit Laurus Private Limited, ICICI Knowledge Park, Turkapally, Shameerpet, Hyderabad-500078, India; 2 Institute of Science and Technology, JNTU, Hyderabad-500072, India

**Keywords:** impurity profile, related substances, sertindole

## Abstract

Sertindole (**1**), an atypical anti-psychotic drug is used for the treatment of schizophrenia. During the laboratory optimization and later during its bulk synthesis the formation of various impurities was observed. The impurities formed were monitored and their structures were tentatively assigned on the basis of their fragmentation patterns in LC-MS. Most of the impurities were synthesized and their assigned constitutions confirmed by co-injection in HPLC. We describe herein the formation, synthesis and characterization of these impurities. Our study will be of immense help to others to obtain chemically pure sertindole.

## Introduction

The safety of a drug product is not only dependent on the toxicological properties of the active drug substance (or API), but also on the impurities formed during the various chemical transformations. Therefore, identification, quantification, and control of impurities in the drug substance and drug product are important parts of drug development for obtaining marketing approval. It is more challenging for an organic chemist to identify the impurities which are formed in very small quantities in a drug substance. Since most of the time it is very difficult to identify and control impurities within acceptable levels in the process, extra purification steps may then be necessary thereby making the process less competitive. More often than not, the syntheses of impurities are not described in the literature which makes it even more difficult for the organic chemist who must then design a synthesis, which is time consuming. The development of a drug substance is incomplete without the identification of an impurity profile involved in the process. Furthermore, it is not mandatory to design synthetic routes for the impurities. Thus, in our study we explored the formation, identification, synthesis and characterization of impurities found in the preparation of sertindole. This study will be of immense help for organic chemists to understand the potential impurities in sertindole synthesis and thereby obtain the pure compound.

**Figure 1:**
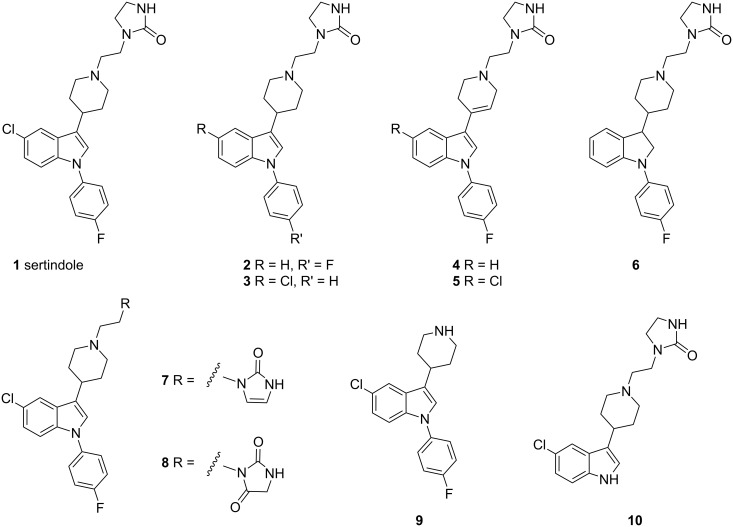
Sertindole (**1**), process related impurities and metabolites.

Sertindole (**1**) (Figure 1)**,** displays broad pharmacological profile and mainly affects dopamine D_2_, serotonin 5-HT_2_ and α_1_-adrenergic receptors [1–5]. It is a potent centrally acting 5-HT_2_ receptor antagonist in vivo and finds application in the treatment of anxiety, hypertension, drug abuse and cognitive disorders. It has been reported to show antipsychotic effect in clinical studies. In contrast to other antipsychotics, sertindole has no associated sedative effects; sedation may add to the cognitive problems inherent in schizophrenia.

**Scheme 1:**
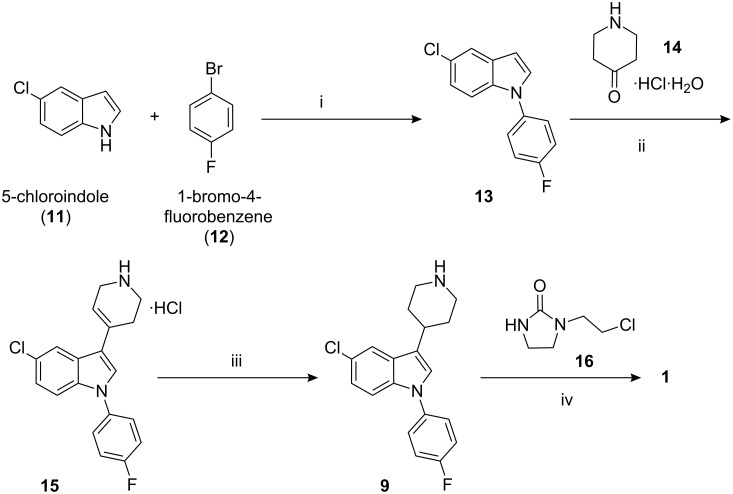
Reagents and conditions: i) K_2_CO_3_, CuBr, ethylenediamine, DMF 130–135 °C; ii) CH_3_COOH, CF_3_COOH, 100–110 °C; iii) PtO_2_/H_2_, methanol, 30–35 °C; iv) K_2_CO_3_, KI, methylisobutyl ketone (MIBK),110–115 °C.

Sertindole is designated chemically as 1-[2-[4-[5-chloro-1-(4-fluorophenyl)-1*H*-indol-3-yl]-1-piperidinyl]ethyl]-2-imidazolidinone. Its literature synthesis (Scheme 1) [1–5] involves the copper catalyzed *N*-arylation of 5-chloroindole (**11**) with 4-fluorobromobenzene (**12**). The product, 5-chloro-1-(4-fluorophenyl)indole (**13**), on treatment with 4-piperidinone hydrochloride monohydrate (**14**) under acidic conditions affords 5-chloro-1-(4-fluorophenyl)-3-(1,2,3,6-tetrahydropyridin-4-yl)-1*H*-indole hydrochloride (**15**)**.** Reduction of **15** in the presence of platinum oxide yields 5-chloro-1-(4-fluorophenyl)-3-(4-piperdinyl)-1*H*-indole (**9**) which on condensation with 1-(2-chloroethyl)imidazolidinone (**16**) in the presence of a base gives sertindole (**1**).

During the laboratory optimization of sertindole (**1**), many process related impurities were identified. The guidelines recommended by ICH state that the acceptable levels for a known and unknown compound (impurity) in the drug should be less than 0.15 and 0.10%, respectively [6]. In order to meet the stringent regulatory requirements, the impurities present in the drug substance must be identified and characterized. Literature reports [5,7–9] include impurities formed due to either over reduction (e.g., **2**, **3** and **6**) [5,7], incomplete reduction (e.g., **4** and **5**) [5,8] or due to incomplete alkylation (e.g., **9** and **10**) [5,7]. However, no synthetic details have been reported. In this context, the present study describes identification, synthesis and characterization of impurities formed during sertindole synthesis.

## Results and Discussion

**Scheme 2:**
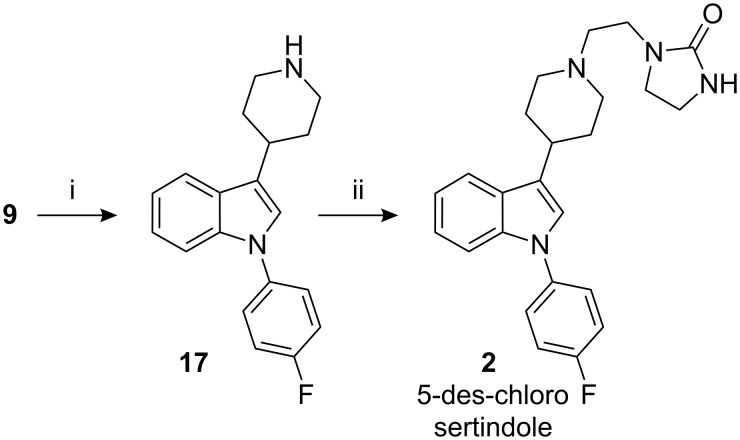
Reagents, conditions (and yields): i) (a) pH adjusted to 6; (b) Pd/C, HCOONH_4_, AcOH, MeOH, reflux (77.6%); ii) **16**, K_2_CO_3_, KI, MIBK, reflux (44.5%).

During the catalytic hydrogenation of indole **15,** formation of 0.5–1.0% of the des-chloro indole **17** is observed; the level is reduced to less than 0.1% during its isolation and purification. It is necessary to remove the impurity at this stage because after condensation with imidazolidinone **16**, it is difficult to remove the des-chloro sertindole **2**, from sertindole, without significant loss in yield. The impurity **2** was prepared by palladium catalyzed hydrogenation of indole **9** and the des-chloro indole **17** formed was condensed with imidazolidinone **16**, under Finkelstein conditions (Scheme 2).

**Scheme 3:**
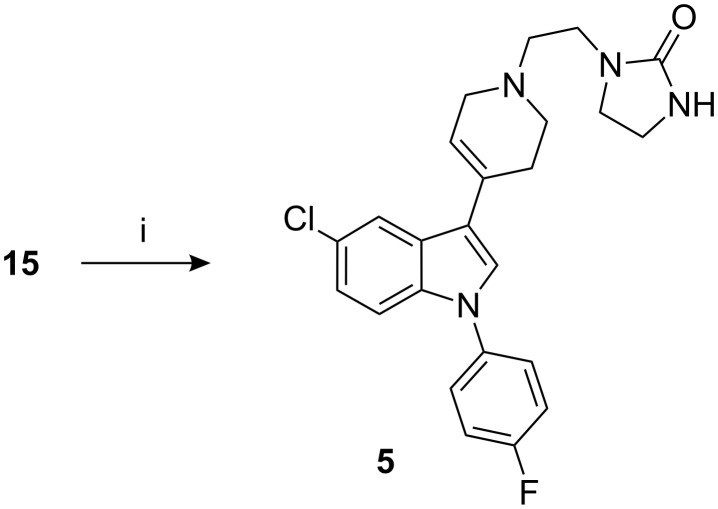
Reagents, conditions (and yields): i) **16**, K_2_CO_3_, KI, MIBK, reflux (73.9%).

The platinum catalyzed reduction of indole **15** is a critical reaction, whilst a prolonged reaction time leads to dehalogenated product **2**, termination without complete reduction leads to indole **9** contaminated with **15**. This contaminated material upon condensation with imidazolidinone **16** results in sertindole (**1**) contaminated with **5**. The degree of contamination was 0.02–0.10% [10]. It is difficult to remove the impurity **5** from sertindole (**1**). Indole **5** was prepared by condensation of **15** with 1-(2-chloroethyl)imidazolidinone (**16**) in the presence of base (Scheme 3).

**Scheme 4:**
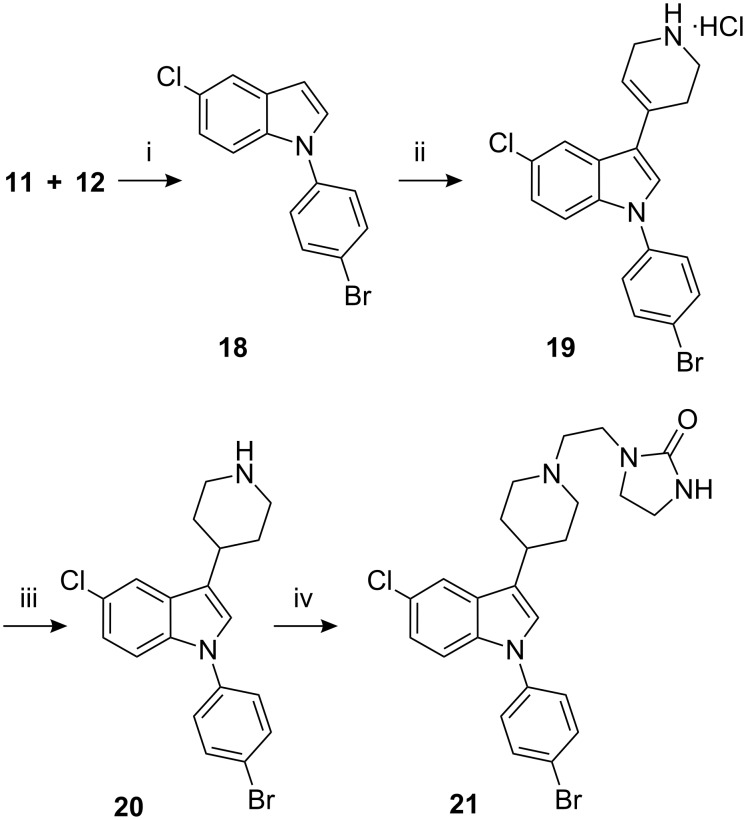
Reagents, conditions (and yields): i) Cs_2_CO_3_, DMF, 130–135 °C; ii) **14**, CH_3_COOH, CF_3_COOH, 100–105 °C (43.4% from **11**); iii) (a) pH adjusted to 6–7; (b) H_2_, PtO_2_, MeOH, AcOH, 30–35 °C; (c) crystallization (24.7%); iv) **16**, K_2_CO_3_, KI, MIBK, reflux (65% yield).

During the *N*-arylation of 5-chloroindole (**11**) with 1-bromo-4-fluorobenzene (**12**), formation of traces of 5-chloro-1-(4-bromophenyl) indole (**18**) along with the desired indole **13** was observed. A detail study of this coupling reaction revealed significant formation of this undesired indole in the absence of a transition metal, especially when cesium carbonate was used for the coupling reaction. Sertindole (**1**) synthesized from this contaminated material was found to contain impurity **21** at levels of 0.05–0.25% [10]. In order to quantify and limit the impurities in the final drug substance, indole **18** was converted to the corresponding *N*-(4-bromophenyl)-impurity **21** (Scheme 4). The catalytic hydrogenation of indole **19** proved to be a difficult reaction as significant formation of the dehalogenated products was observed, repeated recrystallization from MeOH afforded pure indole **20**.

**Scheme 5:**
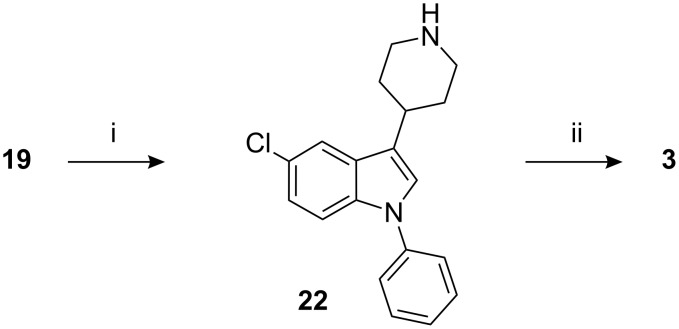
Reagents, conditions (and yields): i) (a) pH adjusted to 6–7; (b) H_2_, PtO_2_, MeOH, AcOH, 30–35 °C (73% yield); ii) **16**, K_2_CO_3_, KI, MIBK, reflux (52%).

Since dehalogenation was observed during the platinum oxide reduction of the tetrahydropyridinyl moiety in indole **19**, this reaction was utilized to synthesize the des-fluoro sertindole **3**. Thus prolonged hydrogenation of **19** afforded des-fluoro indole **22**, which on condensation with imidazolidinone **16** afforded the desired compound **3** (Scheme 5). The contamination by **3** in sertindole (**1**) was 0.20–0.50% [10].

**Scheme 6:**
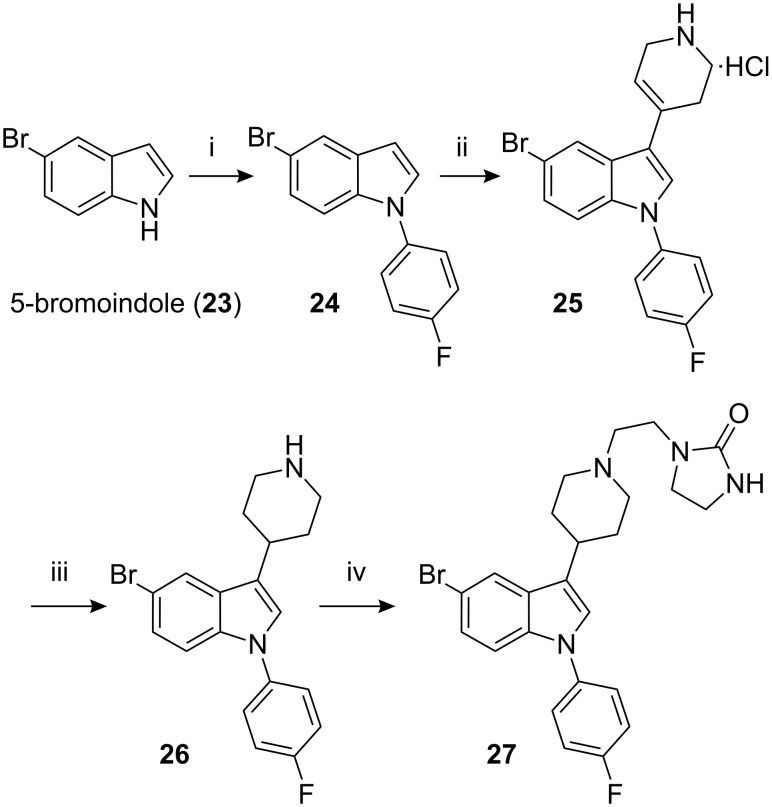
Reagents, conditions (and yields): i) **12**, K_2_CO_3_, Cu(II)Br, ethylenediamine, DMF, 130–135 °C, (51%); ii) **14**, CH_3_COOH, CF_3_COOH, 100–105 °C (57%); iii) (a) pH adjusted to 6–7; (b) PtO_2_/H_2_, MeOH, 30–35 °C; (c) column chromatography (33.3%); iv) **16**, K_2_CO_3_, KI, MIBK, reflux (64.3%).

In some of commercial samples, 5-chloroindole was found to be contaminated with traces of 5-bromoindole (**23**), although the amount of contamination of the bromo analogue **27** in sertindole (**1**) was never more than 0.02%. The bromo analogue of sertindole (**27**) was synthesized from 5-bromoindole (**23**) following the reaction sequence used to synthesize **1** (Scheme 6).

**Scheme 7:**
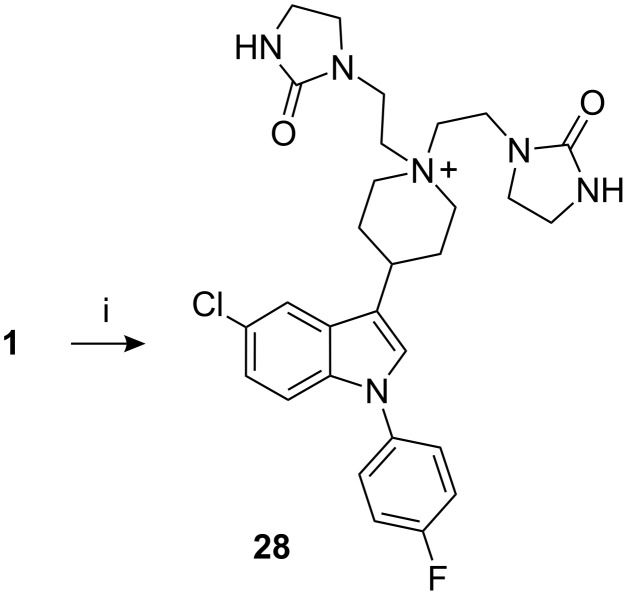
Reagents, conditions (and yield): i) (a) **16**, Et_3_N, NaI, CH_3_CN, reflux; (b) column chromatography (19.1%).

During the *N*-alkylation reaction there is always the probability of dialkylation, hence the dialkylated piperidine **28** was synthesized by prolonged condensation of imidazolidinone **16** with sertindole (**1**)**,** followed by purification by column chromatography (Scheme 7). The contamination by **28** in sertindole (**1**) was 0.25–0.45% [10].

**Scheme 8:**
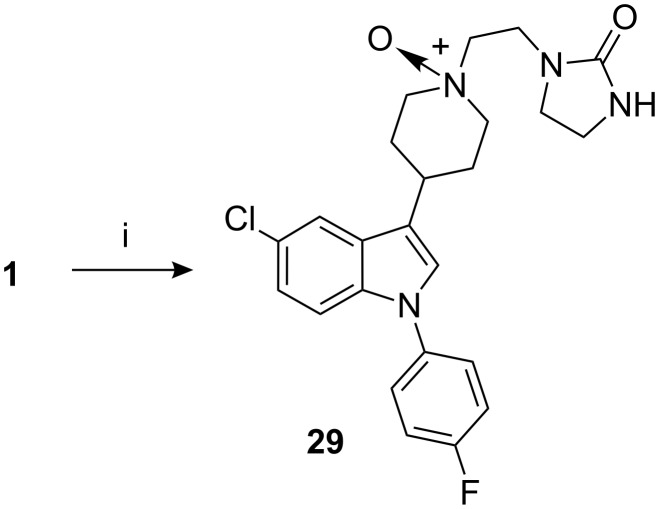
Reagents, conditions (and yield): i) (a) mCPBA, MeOH, 40–45 °C; (b) column chromatography (52.2%).

Sertindole *N*-oxide **29,** a possible contaminant that can be formed by air oxidation, was prepared by oxidation of sertindole (**1**) with *m*-chloroperbenzoic acid (Scheme 8). The contamination by **29** in sertindole (**1**) was <0.05% [10].

## Conclusion

For the better understanding of the synthetic pathway of an active pharmaceutical ingredient (API) it is necessary to identify all the impurities formed/anticipated. In this regard we have synthesized and characterized different potential process-related impurities of sertindole.

## Supporting Information

Experimental procedure (Supporting Information File 1); HPLC chromatograms, ^1^H and ^13^C NMR spectra of compounds **2**, **3**, **5**, **9**, **21**, **27**, **28** and **29** (Supporting Information File 2). HPLC chromatogram containing sertindole (**1**) spiked with process related impurities and LC-MS fragmentation data [10] is included in Supporting Information File 2.

File 1 Full experimental details and characterization data for all new compounds.

File 2 ^1^H and ^13^C NMR spectral data and HPLC chromatograms for all new compounds.

## References

[1] Perregaard J K (1986). Heterocyclic compounds.

[2] Perregaard J K (1987). 1-(4'-fluorophenyl)-3,5-substituted indoles useful in the treatment of psychic disorders and pharmaceutical compositions thereof.

[3] Perregaard J K, Skarsfeldt T (1990). Use of sertindole for the treatment of schizophrenia.

[4] Zanon J, Villa M, Ciardella F (2003). Method for manufacture of sertindole.

[5] Perregaard J, Arnt J, Bøgesø K P, Hyttel J, Sanchez C (1992). J Med Chem.

[6] (2002). ICH guidelines, Q3A (R): Impurities in new drug products: The quality guidelines for active pharmaceutical ingredients related to impurities according to the International Conference of Harmonization.

[7] Pearlstein R A, Vaz R J, Kang J, Chen X L, Preobrazhenskaya M, Shchekotikhin A E, Korolev A M, Lysenkova L N, Miroshnikova O V, Hendrix J (2003). Bioorg Med Chem Lett.

[8] Andersen K, Liljefors T, Gundertofte K, Perregaard J, Bøgesø K P (1994). J Med Chem.

[9] Tzeng T-B, Stamm G, Chu S-y (1994). J Chromatography B.

[R10] 10The degree of formation of the impurities and the HPLC chromatogram included in the Supporting Information File S2, page 29, is for samples of sertindole (**1**) obtained before laboratory optimization.

