# Supplementary material for: Identification and synthesis of impurities formed during sertindole preparation
Source: Beilstein J Org Chem. 2011 Jan 7;7:29–33. doi: 10.3762/bjoc.7.5 (PMC3028988; doi:10.3762/bjoc.7.5)
Supplement: File 1 — Full experimental details and characterization data for all new compounds. [file Beilstein_J_Org_Chem-07-29-s001.pdf]

Supporting Information

for

Identification and synthesis of impurities formed during sertindole preparation

I. V. Sunil Kumar*[1], G. S. R. Anjaneyulu[1] and V. Hima Bindu[2]

Address: [1]Research and Development Centre, Aptuit Laurus Private Limited, ICICI Knowledge Park, Turkapally, Shameerpet, Hyderabad-500078, India and [2]Institute of Science and Technology, JNTU, Hyderabad-500072, India

Email: I. V. Sunil Kumar - sunil.indukuri@aptuitlaurus.com

*Corresponding author

Supporting Information File 1:

Full experimental details and characterization data for all new compounds

General

The ^{1}H and ^{13}C NMR spectra were recorded on a Bruker 300 MHz Avance FT-NMR spectrometer; the chemical shifts were reported in δ ppm relative to either residual solvent or tetramethylsilane. The FT-IR spectra were recorded on Perkin-Elmer 100 FT-IR Spectrophotometer and only prominent peaks are reported. For electrospray ionization

ESI-MS studies a quadrupole mass spectrometer Waters Quatro Micro API was used, whilst an Agilent 6520 accurate-Mass Q-TOF LC/MS system was employed for HRMS studies. HPLC analysis was carried out on Agilent 1200 series or Waters Alliance 2695 systems. All the reagents used were of LR grade and used without further purification. All the anhydrous reactions were carried out under a nitrogen atmosphere. Silica gel (120–200 mesh) was used for column chromatography.

1-[2-[4-[1-(4-Fluorophenyl)-1*H*-indol-3-yl]-1-piperidinyl]ethyl]-2-imidazolidinone (2): To a suspension of oxalate salt of 5-chloro-1-(4-fluorophenyl)-3-(4-piperdinyl)-1*H*-indole **9** (20 g, 47.8 mmol) in CH_2Cl_2 (200 mL), water (200 mL) was added and the mixture stirred for 5 min at 25–30 °C. The pH was adjusted to 10.7 by the dropwise addition of 10% aq NaOH solution (w/w, ca. 75 mL). The clear biphasic system was stirred for 15 min and then the layers were separated. The aqueous phase was extracted with CH_2Cl_2 (50 mL) and the combined organic phases washed with water (50 mL × 2), dried (Na_2SO_4) and concentrated under reduced pressure. To the residue, MeOH (300 mL) was added and the mixture stirred until a clear solution was obtained. The pH was adjusted to 6.0 by the dropwise addition of AcOH (ca. 5 mL). The catalyst, 5% Pd/C (4.0 g; 50% wet), and $HCOONH_4$ (10 g, 158.7 mmol) were added and the mixture heated under reflux until TLC revealed the completion of the reaction. The reaction mixture was cooled to room temperature (rt), the catalyst removed by filtration through a bed of Celite and then washed with MeOH (15 mL). The MeOH was removed by distillation to afford 1-(4-fluorophenyl)-3-(4-piperdinyl)-1*H*-indole **17** (10.9 g, 77.6%) as an off-white solid, with a HPLC purity of 94.5%. Mass spectral data (ESI-MS) showed molecular ion peak (M+H) at *m/z* 295 and the absence of a chlorine isotopic pattern.

To a mixture of the crude indole **17** (7.0 g, 23.8 mmol) and 1-(2-chloroethyl)-2-imidazolidinone **16** (5.3 g, 35.7 mmol) in methyl isobutyl ketone (MIBK) (100 mL) under an inert atmosphere at 25-30 °C, was added KI (0.17 g, 1.0 mmol) and anhydrous K_2CO_3 (5.9 g, 42.8 mmol). The mixture was heated under reflux for 5 h, when analytical TLC revealed the amount of indole **17** remaining was below 2%. The reaction mixture was concentrated under reduced pressure and the residue dissolved in CH_2Cl_2 (70 mL). The organic layer was washed with water (70 mL) and the aqueous phase was extracted with CH_2Cl_2 (35 mL). The combined organic phase was washed with water (35 mL), dried (Na_2SO_4) and concentrated under reduced pressure. The residue was dissolved in acetone (150 mL), carbon PS-133 (1.0 g) added, the solution heated under reflux for 15 min and then cooled to rt. Carbon was removed by filtration through a bed of Celite and the bed washed with acetone (10 mL × 2). The filtrate was concentrated under reduced pressure until approx 20 mL of the solution remained and the resulting slurry stirred for 1 h. The precipitate was filtered, washed with acetone (5 mL × 3) and dried under vacuum at 50–55 °C until the loss on drying (LoD) was <0.5%. Des-chloro sertindole **2** (4.3 g, 44.5%, HPLC purity 96.1%) was obtained as a pale yellow solid. IR (KBr): 3222, 2934, 1681, 1510, 1458, 1284 cm^{-1}. ^1H NMR (DMSO-d_6): δ 7.64 (d, *J* 7.2, 1H), 7.59–7.57 (m, 2H), 7.45 (d, *J* 8.1, 1H), 7.37 (s, 1H), 7.37–7.35 (m, 2H), 7.17–7.14 (m, 1H), 7.08 (m, 1H), 6.21 (brs, 1H), 3.39–3.36 (m, 2H), 3.21–3.18 (m, 2H), 3.16 (t, *J* 6.6, 2H), 2.99 (d, *J* 11.4, 2H), 2.78 (m, 1H), 2.41 (t, *J* 6.6, 2H), 2.09 (t, *J* 11.4, 2H), 1.95 (d, *J* 11.1, 2H), 1.70–1.66 (m, 2H). ^{13}C NMR (DMSO-d_6): δ 162.5, 160.4 (d, *J* 241.5), 136.0 (d, *J* 2.3), 135.9, 128.2, 126.1 (d, *J* 8.3), 124.5, 122.7, 122.4, 120.1, 119.7, 116.8 (d, *J* 22.5), 110.5, 56.5, 54.2, 45.3, 37.9, 33.3, 33.0. ESI-HRMS: *m/z* 407.2284 ($[MH]^+$, $C_{24}H_{28}FN_4O$ calcd. 407.2241).

1-[2-[4-[5-Chloro-1-(4-fluorophenyl)-1*H*-indol-3-yl]-1,2,3,6-tetrahydro-1-pyridyl]ethyl]-2-imidazolidinone (5): To the mixture of crude indole **15** (50.0 g, 137 mmol) and imidazolidinone **16** (30.8 g, 207 mmol) in MIBK (750 mL) at 25–30 °C, was added KI (1.0 g, 6.0 mmol) and anhydrous K_2CO_3 (33.6 g, 243 mmol). The mixture was heated under reflux for 28 h under inert atmosphere, when analytical TLC revealed the amount of indole **15** remaining was below 2%. The reaction mixture was concentrated under reduced pressure and then partitioned between CH_2Cl_2 (500 mL) and water (500 mL). The layers were separated and the organic layer was washed with water (500 mL) and dried (Na_2SO_4). Carbon PS-133 (2.5 g) was added to the organic layer and stirred for 30 min at rt. Carbon was removed by filtration through a bed of Celite and the bed washed with CH_2Cl_2 (25 mL × 2). The solvent was removed under reduced pressure and acetone (100 mL) added to the residue. The resulting slurry was stirred for 1 h at rt, the solids removed by filtration and washed with acetone (10 mL × 3). The wet material (45.0 g) obtained was dried under vacuum at 50–55 °C to afford anhydro-sertindole **5** (44.6 g, 73.9%) as an off-white solid. To the crude material (20 g), MeOH (1200 mL) was added and the solution stirred at 50–55 °C for 30 min. The homogeneous solution was allowed to cool slowly to rt and then to 0–5 °C. The slurry was further stirred at 0–5 °C for 30 min and the solids removed by filtration and washed with ice-cold MeOH (10 mL × 2). The solvent wet material (20 g) was dried under vacuum at 50–55 °C until the LoD was <0.5% to afford anhydro-sertindole **5** (16.1 g, HPLC purity 97.8%; eluted at 0.93 relative retention time w.r.t. sertindole). IR (KBr): 3246, 2899, 2824, 1687, 1512, 1458, 1278, 774 cm^{-1}. ^{13}C NMR (DMSO-d_6): δ 162.5, 160.9 (d, *J* 240.8), 135.1 (d, *J* 3.0), 135.1, 128.7, 128.0, 127.4, 126.7 (d, *J* 8.3), 125.7, 122.9, 120.6, 120.2, 117.8, 117.0 (d, *J* 23.3), 112.4, 55.9, 53.1, 50.1, 45.1, 40.9, 37.8, 28.9. ^{1}H NMR (DMSO-d_6): δ 7.89 (d, *J* 2.1, 1H), 7.72 (s, 1H), 7.63-7.58

(m, 2H), 7.44 (d, J 9.0, 1H), 7.40–7.37 (m, 2H), 7.20 (dd, J 2.1 and 9.0, 1H), 6.23 (brs, 1H), 6.17 (brs, 1H), 3.40-3.34 (m, 2H), 3.23–3.15 (m, 6H), 2.65 (t, J 5.4, 2H), 2.52–2.47 (m, 4H). ^{13}C NMR (DMSO-d_6): δ 162.5, 160.9 (d, J 240.8), 135.1 (d, J 3.0), 135.1, 128.7, 128.0, 127.4, 126.7 (d, J 8.3), 125.7, 122.9, 120.6, 120.2, 117.8, 117.0 (d, J 23.3), 112.4, 55.9, 53.1, 50.1, 45.1, 40.9, 37.8, 28.9. ESI-HRMS: *m/z* 439.1716 ($[MH]^+$, $C_{24}H_{25}ClFN_4O$ calcd. 439.1696), 441.1688 $[MH+2]^+$.

1-[2-[4-[5-Chloro-1-(4-bromophenyl)-1*H*-indol-3-yl]-1-piperidinyl]ethyl]-2-imidazolidinone (21): Anhydrous Cs_2CO_3 (215.0 g, 659.8 mmol) was added to a stirred solution of 5-chloroindole **11** (50.0 g, 330 mmol) and 1-bromo-4-fluorobenzene **12** (144.4 g, 825 mmol) in anhydrous DMF (100 mL). The temperature was raised to 130–135 °C and maintained at this temperature for 20 h, when analytical TLC revealed the amount of remaining 5-chloroindole **11** was below 2%. The reaction mixture was allowed to cool to 55–60 °C and toluene (200 mL) and water (100 mL) were added. The mixture was stirred for 15 min and then the pH adjusted to 2.0–3.0 by the addition of conc HCl (ca. 50 mL). The layers were separated and the aqueous layer was extracted with toluene (100 mL). The combined organic phase was washed with water (100 mL) and dried (Na_2SO_4). Carbon PS-133 (2.5 g) was added to the organic layer and stirred for 30 min at rt. Carbon was removed by filtration through a bed of Celite and the bed washed with toluene (25 mL × 2). The filtrate was concentrated under reduced pressure and the residue **18** (78 g) obtained was used in the next step without any further purification. The residue was approx. 60% pure by HPLC and the assigned constitution was confirmed by LC-MS which displayed molecular a molecular ion peak at *m/z* 305 with the characteristic chlorine-bromine isotopic abundance pattern.

To a solution of the crude indole **18** (65 g) in AcOH (650 mL), was added 4-piperidone **14** (181.7 g) and the temperature of the reaction mixture raised to 70–75 °C. Trifluoroacetic acid (430 mL) was added dropwise over a 45 min period and the temperature raised to 100–105 °C. After maintaining at this temperature for another 1 h, analytical TLC showed that the amount of indole **18** remaining was below 1%. The reaction mixture was allowed to cool to rt and conc HCl (720 mL) added dropwise, when precipitation of a white colored solid was observed. Acetone (850 mL) was added to reaction mixture and the resulting slurry stirred for an hour at rt. The solids were removed by filtration and washed with acetone (20 mL × 3). The solvent wet material (63.0 g) obtained was dried under vacuum at 50–55 °C to afford indole **19** (50.8 g, 43.4% yield from 5-chloroindole **11**; HPLC purity: 99.7%) as an off-white solid.

To a slurry of indole **19** (15.0 g, 35.4 mmol) in a mixture of CH_2Cl_2 (300 mL) and water (150 mL), 20% aq NaOH solution (w/w, ca. 80 mL) was added dropwise to give a pH of 11–12. The clear biphasic system was stirred for 30 min and the layers separated. The aqueous layer was extracted with CH_2Cl_2 (50 mL). The combined organic phase was washed with water (75 mL), dried (Na_2SO_4) and concentrated under reduced pressure. To the residue obtained, was added MeOH (500 mL) and the pH adjusted to 6.0–7.0 by the dropwise addition of AcOH (ca. 4 mL). Carbon PS-133 (0.5 g) was added and the mixture stirred for 60 min at rt. Carbon was removed by filtration through a bed of Celite and the bed washed with MeOH (15 mL × 2). The filtrate was transferred to an autoclave under an inert atmosphere and PtO_2 (454 mg, 2 mmol) added. Hydrogen at a pressure of 3.8–4.2 Kg/cm^2 was applied and the reaction mixture stirred at 30–35 °C. The progress of the reaction was monitored by HPLC. After 32 h <2% of the starting material was present. The H_2 pressure was released and the catalyst removed by filtration, washed with MeOH (5 mL

× 4). The MeOH was removed under reduced pressure and then co-evaporated with EtOAc (20 mL). EtOAc (30 mL) was added to the residue and the resulting slurry stirred for an hour at rt. The solids were removed by filtration, washed with EtOAc (5 mL × 2) and dried under vacuum at 50–55 °C. Two crystallizations from MeOH (24 mL) afforded indole **20** (3.4 g, 24.7% yield; HPLC purity: 93.6%) as a pale yellow solid. The mass spectral data showed the characteristic chlorine-bromine ion abundance pattern.

A mixture of indole **20** (3.0 g, 7.7 mmol), imidazolidinone **16** (1.2 g, 8.1 mmol), KI (30 mg, 0.18 mmol) and anhydrous K_2CO_3 (1.38 g, 10 mmol) in MIBK (45 mL) was heated under reflux for 8 h under an inert atmosphere, when analytical TLC revealed the amount of indole **20** remaining was below 2%. The reaction mixture was allowed to cool to 50–55 °C and the MIBK completely removed under reduced pressure. Dichloromethane (20 mL) and water (20 mL) were added to the residue and the mixture was stirred for 30 min. The layers were separated, the organic layer was washed with water (20 mL) and dried (Na_2SO_4). The solvent was removed under reduced pressure, co-evaporated with acetone (5 mL) and acetone (10 mL) was added to the residue. The resulting slurry was stirred for 1 h at rt, the solid removed by filtration and washed with acetone (2 mL × 3). The solvent wet material (2.9 g) obtained was dried under vacuum at 50–55 °C to afford *N*-(4-bromophenyl) impurity **21** (2.5 g, 65% yield, HPLC purity: 96.2%) as an off-white solid. IR (KBr): 3213, 3090, 2839, 1683, 1589, 1492, 1458 cm^{-1}. ^{1}H NMR (DMSO-d_6): δ 7.72–7.63 (m, 3H), 7.54–7.52 (m, 3H), 7.50 (s, 1H), 7.16 (dd, *J* 2.1 and 8.7, 1H), 6.21 (brs, 1H), 3.39–3.34 (m, 2H), 3.21–3.14 (m, 4H), 2.97 (d, *J* 11.1, 2H), 2.77 (m, 1H), 2.40 (t, *J* 6.6, 2H), 2.08 (t, *J* 11.1, 2H), 1.93 (d, *J* 12.0, 2H), 1.68–1.63 (m, 2H). ^{13}C NMR (DMSO-d_6): δ 162.5, 138.4, 134.0, 133.0, 129.7, 125.9, 124.9, 112.8, 112.7, 119.0, 112.4, 56.5, 54.1,

45.3, 37.9, 32.9, 32.8. ESI-HRMS: *m/z* 501.1057 ($[MH]^+$, $C_{24}H_{27}BrClN_4O$ calcd. 501.1051), 503.1039 $[MH+2]^+$, 505.1016 $[MH+4]^+$.

1-[2-[4-(5-Chloro-1-phenyl-1*H*-indol-3-yl)-1-piperidinyl]ethyl]-2-imidazolidinone (3): Indole **19** (15.0 g, 35.4 mmol) was neutralized with 20% aq NaOH solution as described above. The residue obtained was dissolved in MeOH (500 mL) and the pH adjusted to 6.0–7.0 by the dropwise addition of AcOH (ca. 4 mL). After carbon PS-133 (0.5 g) treatment, the reaction mass was hydrogenated in an autoclave in the presence of catalytic amount of PtO_2 (454 mg, 2 mmol) at 30–35 °C and at a pressure of 6–7 Kg/cm^2. After 24 h of hydrogenation, analytical TLC revealed the completion of the reaction.The H_2 pressure was released, the catalyst removed by filtration and washed with MeOH (5 mL × 4). MeOH was completely removed under reduced pressure. Ethyl acetate (30 mL) was added to the residue, the resulting slurry stirred for an hour at 40–45 °C and then allowed to cool to rt. The solid was filtered and washed with EtOAc (5 mL × 2). The solvent wet material (10.4 g) was dried under vacuum at 50–55 °C until the LoD was < 1% to afford indole **22** (8.0 g, 73% yield) as a pale yellow solid. The mass spectral data (ESI-MS) showed the absence of bromine.

A mixture of indole **22** (5.0 g, 16.1 mmol), imidazolidinone **16** (2.8 g, 18.8 mmol), KI (100 mg, 0.6 mmol) and anhydrous K_2CO_3 (3.2 g, 23.2 mmol) in MIBK (75 mL), was heated under reflux for 24 h under an inert atmosphere, when analytical TLC revealed the amount of indole **22** remaing was below 2%. The reaction mixture was allowed to cool to 50–55 °C and the MIBK completely removed under reduced pressure. Dichloromethane (40 mL) and water (40 mL) were added to the residue and the mixture stirred for 30 min. The layers were separated and the aqueous layer was extracted with CH_2Cl_2 (40 mL). The

combined organic phase was washed with water (40 mL) and dried (Na_2SO_4). The solvent was removed under reduced pressure, co-evaporated with acetone (5 mL × 2) and acetone (50 mL) was added to the residue. The resulting slurry was stirred for 1h at rt, the solid removed by filtration and washed with acetone (5 mL × 3). The acetone wet material (3.9 g) was dried under vacuum at 50–55 °C to afford des-fluoro sertindole **3** (3.7 g, 52% yield, HPLC purity: 95.0%) as a pale yellow solid. Crystallization from a mixture of MeOH-acetone gave material with an HPLC purity of 95%. To a suspension of indole **3** (3.0 g) in MeOH (15 mL) at 50–55 °C, was added acetone (80 mL). The clear solution formed was maintained at 50–55 °C for an hour and then allowed to cool to rt. The crystals formed were filtered, washed with acetone (2 mL × 3) and dried under vacuum at 50–55 °C to afford indole **3** (2.2 g, 73% recovery, HPLC purity: 95%). IR (KBr): 3211, 2940, 1698, 1595, 1501 cm^{-1}. ^{13}C NMR (DMSO-d_6): δ 162.5, 139.2, 130.2, 134.2, 129.5, 126.7, 126.1, 124.7, 124.0, 122.6, 122.2, 118.9, 112.4, 56.5, 54.1, 45.3, 41.0, 33.0, 37.9, 32.9. ^1H NMR (DMSO-d_6): δ 7.69 (d, *J* 2.1, 1H), 7.54–7.52 (m, 4H), 7.51 (d, *J* 9.0, 1H), 7.49 (s, 1H), 7.36 (m, 1H), 7.15 (dd, *J* 2.1 and 9.0, 1H), 6.21 (brs, 1H), 3.39-3.31 (m, 2H), 3.21-3.18 (m, 2H), 3.15 (t, *J* 6.9, 2H), 2.97 (d, *J* 11.4, 2H), 2.76 (m, 1H), 2.40 (t, *J* 6.9, 2H), 2.08 (t, *J* 11.4, 2H), 1.93 (d, *J* 11.1, 2H), 1.69–1.64 (m, 2H). ^{13}C NMR (DMSO-d_6): δ 162.5, 139.2, 134.2, 130.2, 129.5, 126.7, 126.0, 124.7, 124.0, 122.6, 122.2, 118.9, 112.4, 56.5, 54.1, 45.3, 41.0, 37.9, 33.0, 32.9. ESI-HRMS: *m/z* 423.1965 ($[MH]^+$, $C_{24}H_{28}ClFN_4O$ calcd. 423.1946), 425.1939 $[MH+2]^+$.

1-[2-[4-[5-Bromo-1-(4-fluorophenyl)-1*H*-indol-3-yl]-1-piperidinyl]ethyl]-2-imidazolidinone (27): Under an inert atmosphere, anhydrous K_2CO_3 (38.5 g, 278.6 mmol) was added to a stirred solution of 5-bromoindole **23** (40.0 g, 204 mmol), 4-

fluorobromobenzene **12** (92 g, 526 mmol), Cu(II)Br (1.8 g, 8.06mmol), ethylenediamine (2.96 g, 49.3 mmol) in anhydrous DMF (80 mL). The temperature was raised to 130–135 °C and maintained at this temperature for another 48 h, when analytical TLC revealed the amount of 5-bromoindole **23** remaining was below 3%. The reaction mixture was allowed to cool to 55–60 °C and toluene (200 mL) and water (150 mL) were added. The insoluble material was removed by filtration and the layers separated. Water (100 mL) was added to the organic later and the pH adjusted to 2.0–3.0 by the addition of conc HCl (ca. 5 mL). The layers were separated and the organic layer was sequentially washed with water (100 mL) and 10% aq $(NH_4)_2SO_4$ solution (100 mL × 2), and then dried (Na_2SO_4). The solvent was removed under reduced pressure and the residue co-evaporated with MeOH (20 mL × 2). To the residue (78 g), MeOH (100 mL) was added and the mixture stirred for 1 h at 25–30 °C, when precipitation was observed. The mixture was cooled to 0–5 °C and the resulting slurry stirred at the same temperature for another hour. The solid was removed by filtration and washed with ice-cold MeOH (10 mL × 3). The solvent wet material (34 g) was dried under vacuum at 50–55 °C for 9 h to afford indole **24** (32 g, 51%) as a white solid. Its mass spectrum (ESI-MS) displayed the required molecular ion peak at *m/z* 305 and the characteristic bromine isotopic abundance pattern.

A mixture of indole **24** (27.5 g, 94.8 mmol), 4-piperidone **14** (17.3 g, 112.9 mmol), trifluoroacetic acid (82.5 mL) and AcOH (82.5 mL) was stirred at 100–105 °C for 1 h when analytical TLC revealed the amount of indole **24** remaing was below 1%. The reaction mixture was allowed to cool to 50–55 °C, the solvent completely removed under reduced pressure and the residue co-evaporated once with acetone (27.5 mL). To the resulting light-brown solid, acetone (165 mL) was added. The solution was stirred, 6N aq HCl (27.5 mL) added over a 20 min period and the resulting slurry stirred at rt for another 2 h. The

solid was removed by filtration and washed with acetone (10 mL × 3). The solvent wet material (29.3 g) was dried under vacuum at 50–55 °C to afford indole **25** (22.0 g, 57% yield) as a pale yellow solid.

To a slurry of indole **25** (20.0 g, 49.1 mmol) in a mixture of CH_2Cl_2 (500 mL) and water (240 mL), 10% aq NaOH solution (w/w, ca. 40 mL) was added dropwise to give a pH of 11–12, . The clear biphasic system was stirred for 30 min and the layers separated. The aqueous layer was extracted with CH_2Cl_2 (80 mL). The combined organic phases were washed with water (80 mL), dried (Na_2SO_4) and concentrated under reduced pressure. To the residue, was added MeOH (1 Lit) and the pH adjusted to 6.0–7.0 by the dropwise addition of AcOH (ca. 5 mL). Carbon PS-133 (2.0 g) was added and the mixture stirred for 60 min at rt. Carbon was removed by filtration through a bed of Celite and the bed washed with MeOH (25 mL × 2). The filtrate was transferred to an autoclave under an inert atmosphere and PtO_2 (800 mg, 3.52 mmol) added. Hydrogen at a pressure of 3.8–4.2 Kg/cm^2 was applied and the mixture stirred at 30–35 °C. The progress of the reaction was monitored by HPLC and after 4 h <2% of the starting material remained. The H_2 pressure was released, the catalyst removed by filtration and washed with MeOH (15 mL × 2). Carbon PS-133 (1.0 g) was added to the filtrate and the mixture stirred for 30 min at rt. Carbon was removed by filtration through a bed of Celite and the bed washed with MeOH (10 mL × 2). MeOH was removed under reduced pressure, the residue co-evaporated once with EtOAc (20 mL) and then triturated with EtOAc (60 mL) for an hour at rt. The resulting solid was removed by filtration, washed with EtOAc (10 mL × 2), dried under vacuum at 50–55 °C and purified by silica gel column chromatography. Elution with 7–10% MeOH in CH_2Cl_2 (v/v) afforded indole **26** (6.1 g, 33.3% yield; HPLC purity: 97.1%) as

off-white solid. The mass spectral data showed the molecular ion peak at *m/z* 373 [M+H] and a characteristic bromine ion abundance pattern.

A mixture of indole **26** (4.8 g, 12.9 mmol), imidazolidinone **16** (2.24 g, 15.1 mmol), KI (83 mg, 0.5 mmol) and anhydrous K_2CO_3 (2.64 g, 19.1 mmol) in MIBK (75 mL), was heated under reflux for 6 h under an inert atmosphere when analytical TLC revealed the amount of indole **26** remaining was below 2%. The reaction mixture was allowed to cool to 50–55 °C and the MIBK completely removed under reduced pressure. To the residue, CH_2Cl_2 (40 mL) and water (40 mL) were added and the mixture stirred for 30 min. The layers were separated and the organic layer was washed with water (40 mL) and dried (Na_2SO_4). The solvent was removed under reduced pressure and the residue co-evaporated with acetone (20 mL × 2). The resulting sticky solid was triturated for an hour with acetone (40 mL). The solid obtained was removed by filtration and washed with acetone (5 mL × 3). The solvent wet material (4.4 g) was dried under vacuum at 50–55 °C to afford indole **27** (4.0 g, 64.3% yield, HPLC purity: 97.5%) as a pale yellow solid. IR (KBr): 3447, 3237, 2938, 1699, 1508, 1457, 1212 cm^{-1}. ^{1}H NMR (DMSO-d_6): δ 7.83 (d, *J* 1.8, 1H), 7.57 (m, 2H), 7.45 (s, 1H), 7.39 (d, *J* 8.7, 1H), 7.37–7.34 (m, 2H), 7.26 (dd, *J* 1.8 and 8.7, 1H), 6.21 (brs, 1H), 3.39–3.34 (m, 2H), 3.20-3.18 (m, 2H), 3.16 (t, *J* 6.6, 2H), 2.97 (d, *J* 11.1, 2H), 2.77 (m, 1H), 2.40 (t, *J* 6.6, 2H), 2.08 (t, *J* 11.1, 2H), 1.93 (d, *J* 11.1, 2H), 1.71-1.60 (m, 2H). ^{13}C NMR (DMSO-d_6): δ 162.5, 160.7 (d, *J* 241.5), 135.5 (d, *J* 2.3), 134.7, 130.0, 126.3 (d, *J* 8.3), 126.2, 125.2, 122.1, 121.9, 116.9 (d, *J* 22.5), 112.7, 112.6, 56.5, 54.1, 45.3, 37.9, 32.9. ESI-HRMS: *m/z* 485.1349 ($[MH]^+$, $C_{24}H_{27}BrFN_4O$ calcd. 485.1346), 487.1329 $[MH+2]^+$.

5-Chloro-1-(4-fluorophenyl)-3-{1,1-bis[2(2-imidazolidon-1-yl)ethyl]-piperidin-4-yl}-1*H*-indole (28): To a stirred mixture of sertindole (**1**) (5 g, 11.3 mmol) and triethylamine (3 mL) in acetonitrile (35 mL), was added sodium iodide (2.2 g, 14.7 mmol) and imidazolidinone **16** (2.9 g,19.5 mmol). The reaction mixture was heated under reflux. After maintaining the reaction at reflux for 36 h, analytical TLC revealed that only approx 50% conversion had occurred (neither prolonged reaction time nor the addition of excess imidazolidinone **16** had any effect in increasing the yield of product). The reaction mixture was concentrated under vacuum, the residue partitioned between CH_2Cl_2 (35mL) and water (10 mL), stirred for 10 min and the layers separated. The organic layer was washed with water (10 mL), dried (Na_2SO_4) and the solvent completely removed under reduced pressure. The residue (3.8 g, HPLC purity: 49.7%) was purified by silica gel column chromatography. Elution with 10–12% MeOH in CH_2Cl_2 (v/v) afforded the dialkylated piperidine **28** (1.2 g, 19.1% yield; HPLC purity: 96.7%) as an off-white solid. IR (KBr): 3424, 2925, 1682, 1513, 1457, 1217, 794 cm^{-1}. ^1H NMR (DMSO-d_6): δ 7.79 (d, *J* 1.8, 1H), 7.61–7.58 (m, 2H), 7.53 (s, 1H), 7.47 (d, *J* 8.7, 1H), 7.42–7.39 (m, 2H), 7.19 (dd, *J* 1.8 and 8.7, 1H), 6.63 (brs, 1H), 6.62 (brs, 1H), 3.77–3.74 (m, 2H), 3.69–3.65 (m, 6H), 3.62–3.59 (m, 2H), 3.59–3.56 (m, 2H), 3.48–3.44 (m, 4H), 3.31–3.26 (m, 4H), 3.19 (m, 1H), 2.19–2.14 (m, 4H). ^{13}C NMR (DMSO-d_6): δ 162.5, 162.4, 160.8 (d, *J* 238.5), 135.4 (d, *J* 2.3), 134.3, 129.0, 127.2, 126.3 (d, *J* 8.3), 125.0, 122.9, 119.4, 118.8, 117.0 (d, *J* 22.5), 112.4, 60.3, 59.7, 50.8, 45.8, 45.4, 37.8, 29.9, 26.2. ESI-HRMS: *m/z* 553.2494 ($[M]^+$, $C_{29}H_{35}ClFN_6O_2$ calcd. 553.2487), 555.2466 $[M+2]^+$.

1-[2-[4-[5-chloro-1-(4-fluorophenyl)-1*H*-indol-3-yl]-1-oxo-1-piperidinyl]ethyl]-imidazolidin-2-one (29):

A mixture of sertindole (**1**) (5 g, 11.3 mmol) and *m*-chloroperbenzoic acid (2.5 g, 14.5 mmol) in MeOH (150 mL) was stirred at 40–45 °C for 12 h under an inert atmosphere when analytical TLC revealed approx 60–70% conversion (neither prolonged reaction time nor addition of excess mCPBA had any effect in increasing the yield of product). MeOH was completely removed under vacuum and the residue partitioned between CH_2Cl_2 (75 mL) and water (75 mL). The separated organic layer was washed with water (20 mL), dried (Na_2SO_4) and the solvent completely removed under reduced pressure. The residue (7.0 g) was purified by silica gel column chromatography. Elution with 10–15% MeOH in CH_2Cl_2 (v/v) afforded the *N*-oxide **29** (2.7 g, 52.2% yield; HPLC purity: 99.5%) as a pale yellow solid. IR (KBr): 3471, 3291, 2959, 2928, 1674, 1512, 1492 cm^{-1}. ^{13}C NMR (DMSO-d_6): δ 162.5, 160.7 (d, J 242.3), 135.4 (d, J 3.0), 134.5, 129.1, 126.5, 126.3 (d, J 9.0), 124.8, 122.7, 121.0, 116.9 (d, J 22.5), 119.0, 112.3, 67.9, 64.3, 45.4, 38.0, 37.9, 31.2, 27.1. ^1H NMR (DMSO-d_6): δ 7.77 (d, J 2.1, 1H), 7.61–7.56 (m, 2H), 7.53 (s, 1H), 7.44 (d, J 8.7, 1H), 7.40–7.35 (m, 2H), 7.16 (dd, J 2.1 and 8.7, 1H), 6.46 (brs, 1H), 3.60 (t, J 6.3, 2H), 3.40–3.35 (m, 4H), 3.29 (t, J 6.3, 2H), 3.24–3.21 (m, 2H), 3.09 (d, J 10.8, 2H), 2.93 (m, 1H), 2.47–2.41 (m, 2H), 1.81–1.77 (m, 2H). ^{13}C NMR (DMSO-d_6): δ 162.5, 160.7 (d, J 242.3), 135.4 (d, J 3.0), 134.5, 129.1, 126.5, 126.3 (d, J 9.0), 124.8, 122.7, 121.0, 119.0, 116.9 (d, J 22.5), 112.3, 67.9, 64.3, 45.4, 38.0, 37.9, 31.2, 27.1. ESI-MS: *m/z* 457 ($[MH]^+$, $C_{24}H_{26}ClFN_4O_2$ calcd. 456), 459 $[MH+2]^+$.
