# Supplementary material for: Identification and synthesis of impurities formed during sertindole preparation
Source: Beilstein J Org Chem. 2011 Jan 7;7:29–33. doi: 10.3762/bjoc.7.5 (PMC3028988; doi:10.3762/bjoc.7.5)
Supplement: File 2 — 1H and 13C NMR spectral data and HPLC chromatograms for all new compounds. [file Beilstein_J_Org_Chem-07-29-s002.pdf]

Supporting Information

for

Identification and synthesis of impurities formed during sertindole preparation

I. V. Sunil Kumar*[1], G. S. R. Anjaneyulu[1] and V. Hima Bindu[2]

Address: [1]Research and Development Centre, Aptuit Laurus Private Limited, ICICI Knowledge Park, Turkapally, Shameerpet, Hyderabad-500078, India and [2]Institute of Science and Technology, JNTU, Hyderabad-500072, India

Email: I. V. Sunil Kumar - sunil.indukuri@aptuitlaurus.com

*Corresponding author

Supporting Information File 2:

^{1}H and ^{13}C NMR spectral data and HPLC chromatograms for all new compounds

Table of contents

1) HPLC chromatograms, ^1H and ^{13}C NMR spectral data of:

a) Des-chloro sertindole (**2**) 3

b) Des-fluoro sertindole (**3**) 6

c) Anhydro sertindole (**5**) 9

d) Nor-sertindole (**9**) 12

e) 1-(4-Bromophenyl) impurity (**21**) 15

f) 5-Bromo sertindole (**27**) 18

g) Bis-alkylated impurity (**28**) 21

h) Sertindole-N-oxide (**29**) 24

2) HPLC chromatogram – Sertindole spiked with process related impurities 27

3) HPLC chromatogram and LC-MS fragmentation data 29

1H NMR of 0080/2059/003 in DMSO-d6
Date:- 02/06/09 ARNo :SE0509/027
{C:/LKT/Structure elucidation/May2009/94/1}

Des-chloro 2

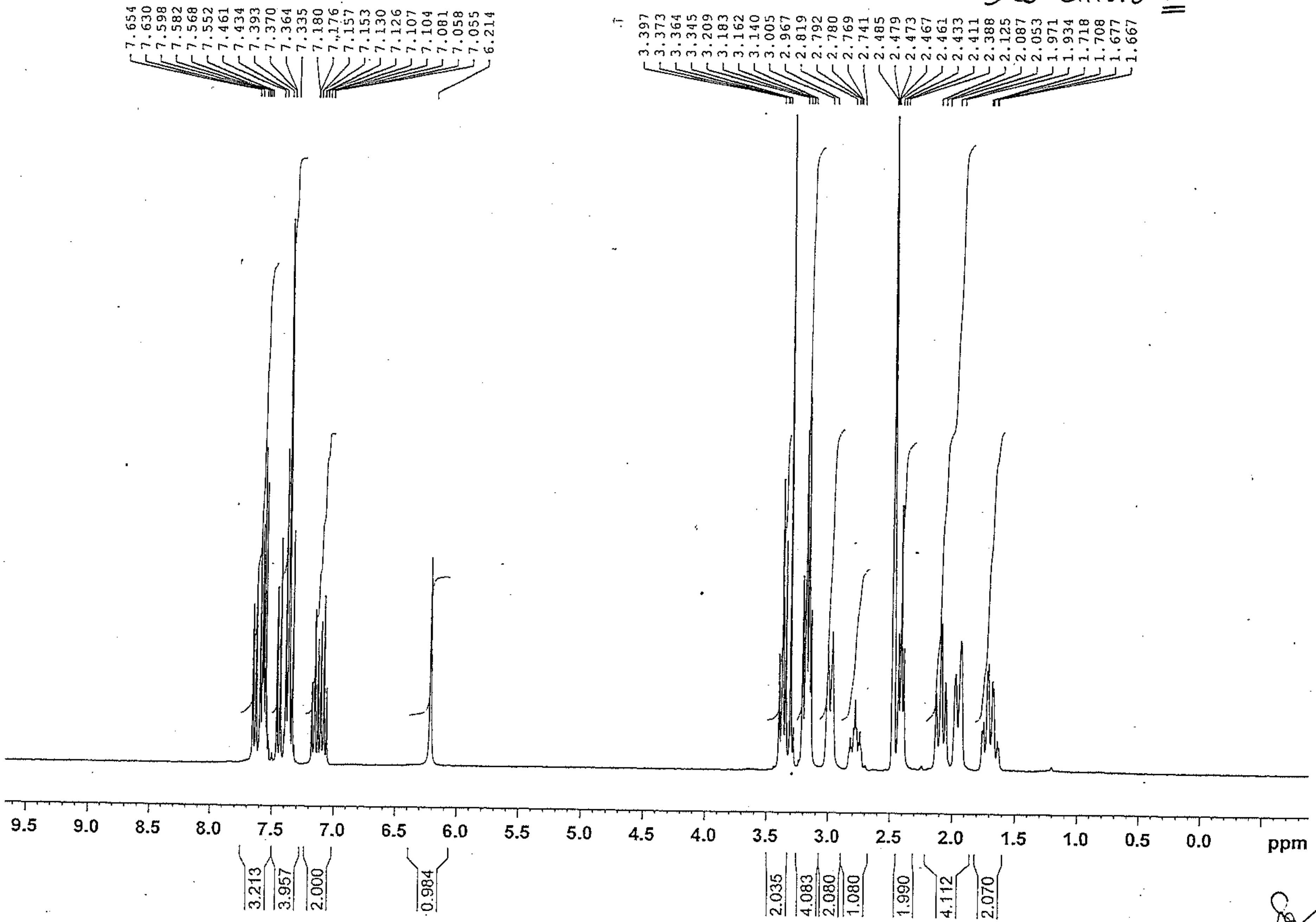

2/06/09

13C NMR of 0080/2059/003 in DMSO-d6
Date:- 02/06/09 ARNo :SE0509/027
{C:/LKT/Structure elucidation/May2009/98/1}

aptuit laurus

Des-Chloro 2

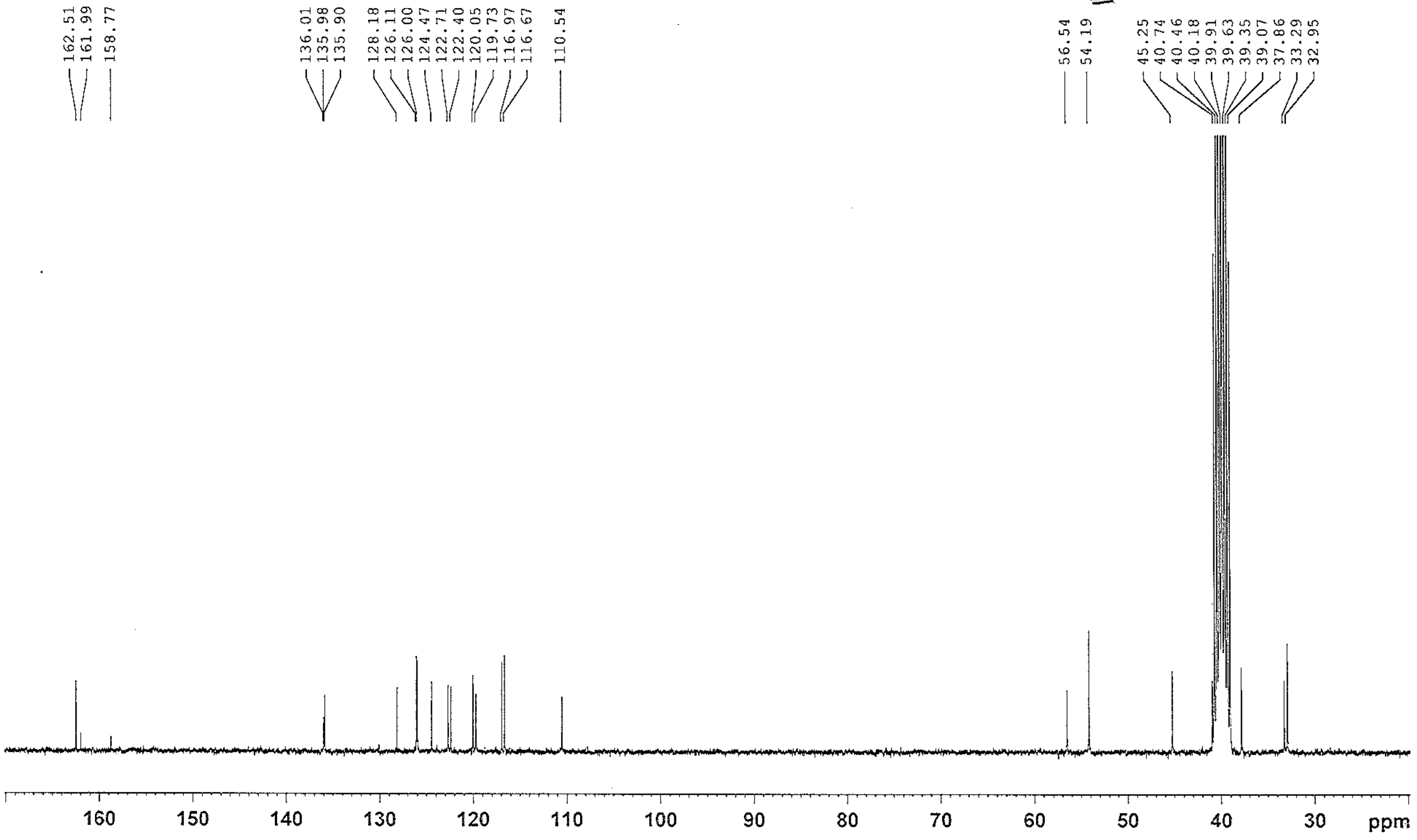

Analyst: Lakshmi Kumar.Tatini

aptuit laurus

Analytical Development

SAMPLE INFORMATION

Sample Name:	0080/ASt-imp/2059/003 pre-1	Instrument Method Id 4820	
Sample Type:	Unknown	Acquired By:	Analyst
Vial:	58	Date Acquired:	7/31/2009 2:33:26 AM IST
Injection #:	1	Date Processed:	7/31/2009 9:49:56 AM IST
Injection Volume:	10.00 ul	Processing Method:	0080_RS
Sample Set Id 5596		Processing Method Id 5731	
Sample Set Name:	300709 01	Proc. Chnl. Descr.:	VWD AU On nm
Project Name: Sertindole_July_09		System Name LL_AD_LC_SYS011	
Result Id 5746	Result Set Id	Software: Empower 2 Software Build 2154	

Auto-Scaled Chromatogram

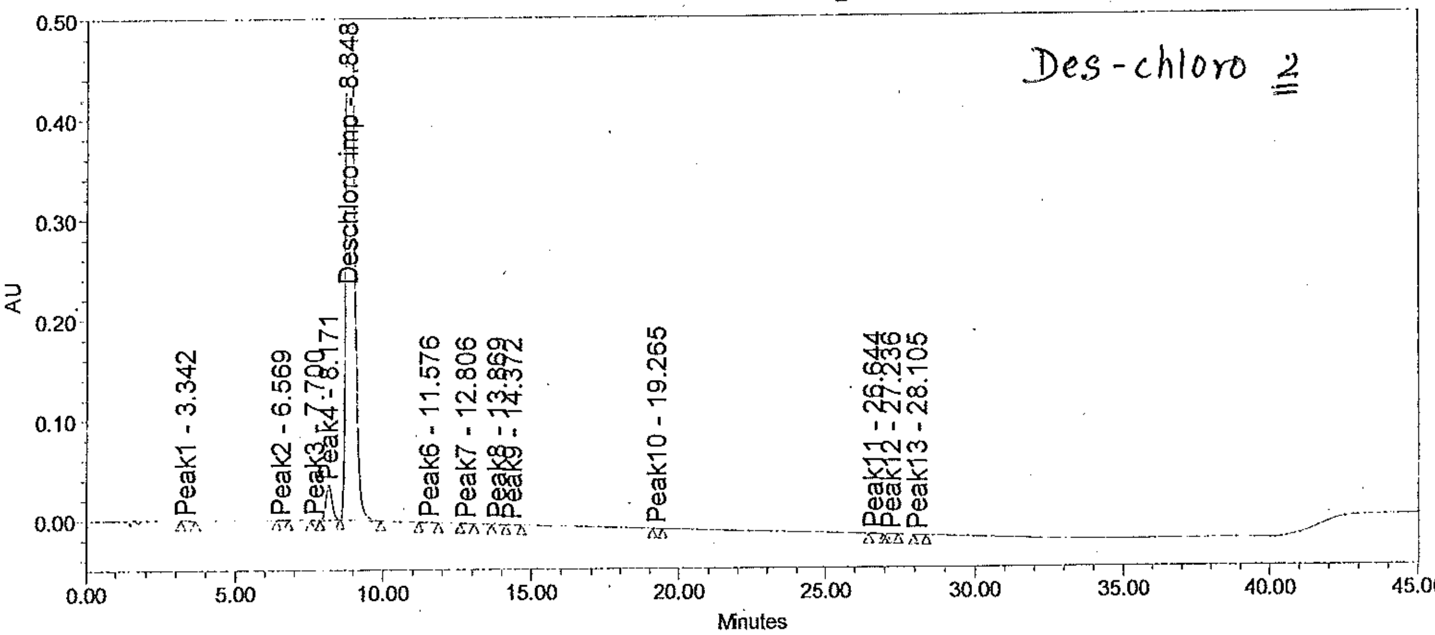

Peak Results

	Name	RT	Area (µV*sec)	% Area	RT Ratio	USP Resolution	USP Tailing	USP Plate Count
1	Peak1	3.34	6067.41	0.04	0.38		1.34	1214
2	Peak2	6.57	1798.65	0.01	0.74	8.46	1.02	2302
3	Peak3	7.70	1917.25	0.01	0.87	3.24	1.04	15365
4	Peak4	8.17	501604.51	3.45	0.92	1.32	1.19	7448
5	Deschloro imp	8.85	13956027.74	96.11	1.00	1.66	1.65	7064
6	Peak6	11.58	9009.57	0.06	1.31	5.56	0.87	6036
7	Peak7	12.81	4204.26	0.03	1.45	2.93	1.04	20843
8	Peak8	13.87	16011.02	0.11	1.57	3.14	1.15	19867
9	Peak9	14.37	6659.43	0.05	1.62	1.30	1.14	25101
10	Peak10	19.27	3827.95	0.03	2.18	14.28	0.94	76063
11	Peak11	26.64	8441.21	0.06	3.01	19.79	1.32	48031
12	Peak12	27.24	2521.73	0.02	3.08	1.44	1.07	121292

1H NMR of 0080/2198/164 in DMSO-d6
Date:- 06/08/09 ARNO :SE0809/06
{C:/LKT/Structure elucidation/Aug-2009/20/1

Des-fluoro 3

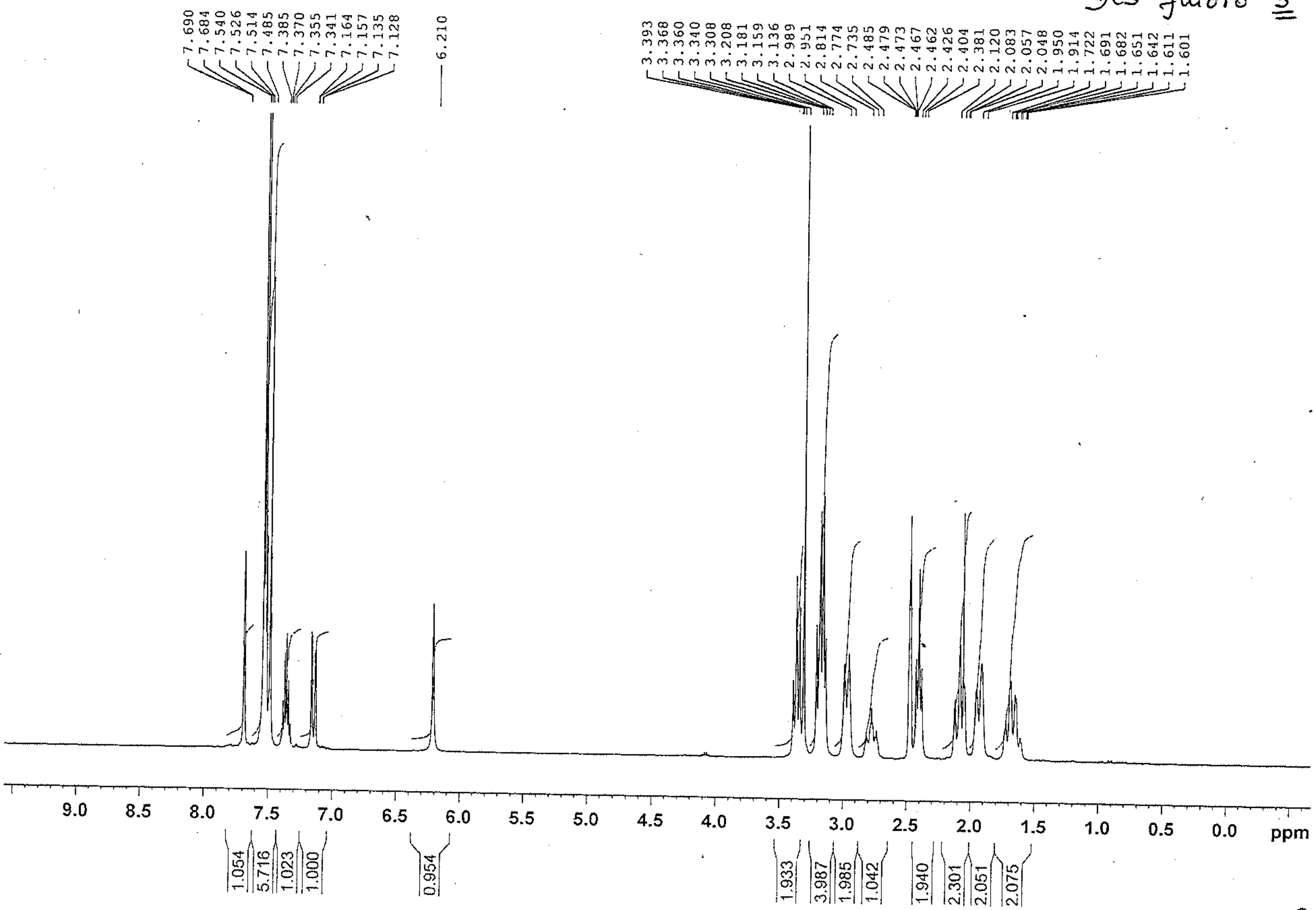

Analyst: Suresh Kumar.K

13C NMR of 0080/A-imp/2198/164 in DMSO-d6
Date:- 14/09/10
{D:/LKT/aptuit laurus/September-2010/178/1}

aptuit laurus

Des-fluoro 3

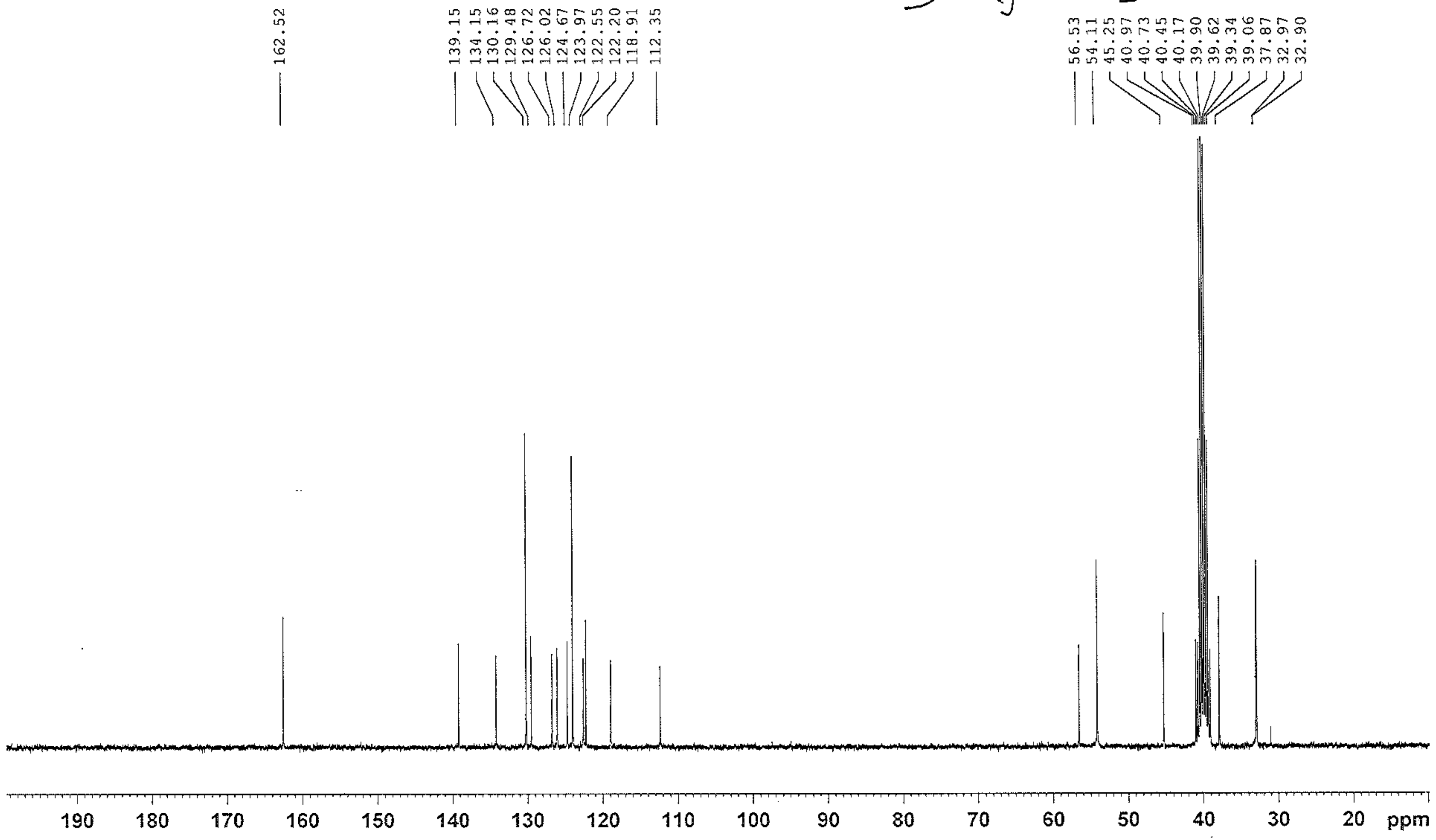

Analyst: Srikanth.M

aptuit laurus

Analytical Development

SAMPLE INFORMATION

Sample Name: 0080/2198/164(Desfluoro)Pre-1
Sample Type: Unknown
Vial: 59
Injection #: 1
Injection Volume: 10.00 ul
Sample Set Id 6733
Sample Set Name: 040809_01
Project Name: Sertindole_July_09
Result Id 7014 Result Set Id

Instrument Method Id 6729
Acquired By: Analyst
Date Acquired: 8/5/2009 10:32:10 AM IST
Date Processed: 8/5/2009 11:55:47 AM IST
Processing Method: 0080_Stage123_RS
Processing Method Id 6996
Proc. Chnl. Descr.:
System Name LL_AD_LC_SYS006
Software: Empower 2 Software Build 2154

Auto-Scaled Chromatogram

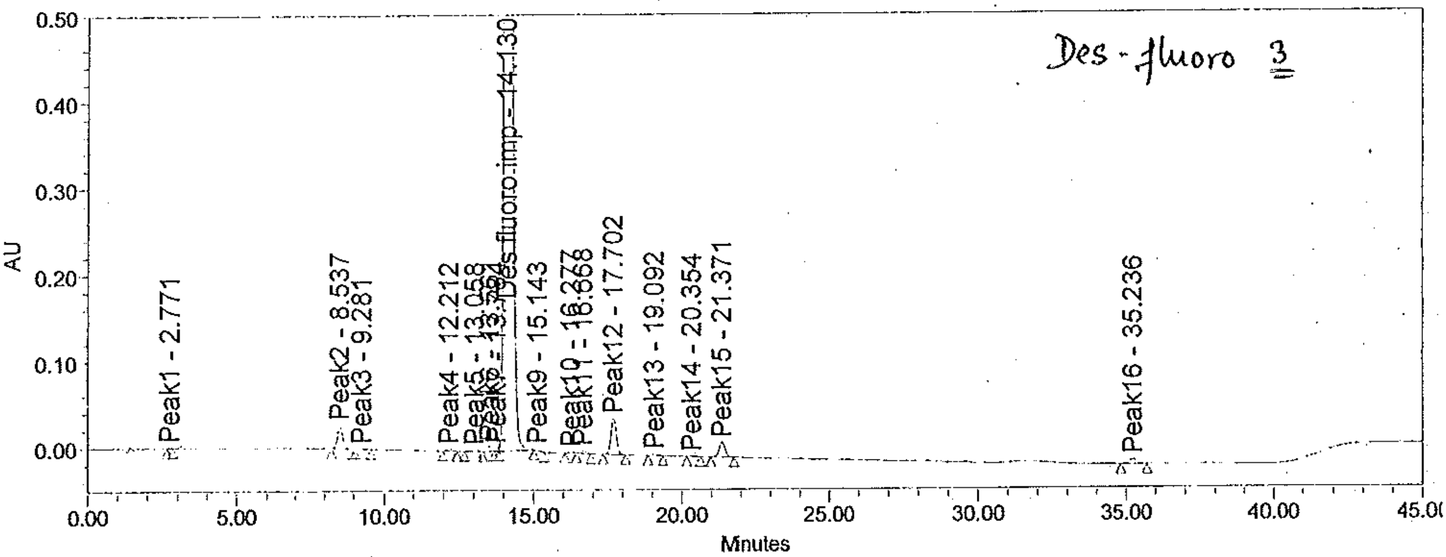

Peak Results

	Name	RT	Area (µV*sec)	% Area	RT Ratio	USP Resolution	USP Tailing	USP Plate Count
1	Peak1	2.77	9367.56	0.03	0.20		0.97	6270
2	Peak2	8.54	315026.63	1.09	0.60	25.76	1.12	12987
3	Peak3	9.28	8431.83	0.03	0.66	2.26	1.41	11458
4	Peak4	12.21	23863.12	0.08	0.86	8.66	1.12	23397
5	Peak5	13.06	29652.15	0.10	0.92	2.05	0.85	12827
6	Peak6	13.56	61613.31	0.21	0.96	1.22		33573
7	Peak7	13.78	23970.56	0.08	0.98			
8	Des fluoro imp	14.13	27411722.68	94.96	1.00		1.35	22506
9	Peak9	15.14	23009.95	0.08	1.07	3.10	1.45	50407
10	Peak10	16.28	16785.55	0.06	1.15	4.05	0.92	51175
11	Peak11	16.67	41175.82	0.14	1.18	1.19	1.15	33289
12	Peak12	17.70	543571.51	1.88	1.25	2.90	1.10	43760
13	Peak13	19.09	24241.61	0.08	1.35	4.03	1.17	50389
14	Peak14	20.35	4448.94	0.02	1.44	3.61	1.39	53655
15	Peak15	21.37	232405.96	0.81	1.51	2.83	1.09	57013

Signature / Date: [illegible] 05/08/09

1H NMR of 0080/1847/031 in DMSO-d6
Date:07/04/09 AR.No:SE0409/01
{C:/LKT/aptuit Laurus/April 2009/21/1}

Anhydro 5

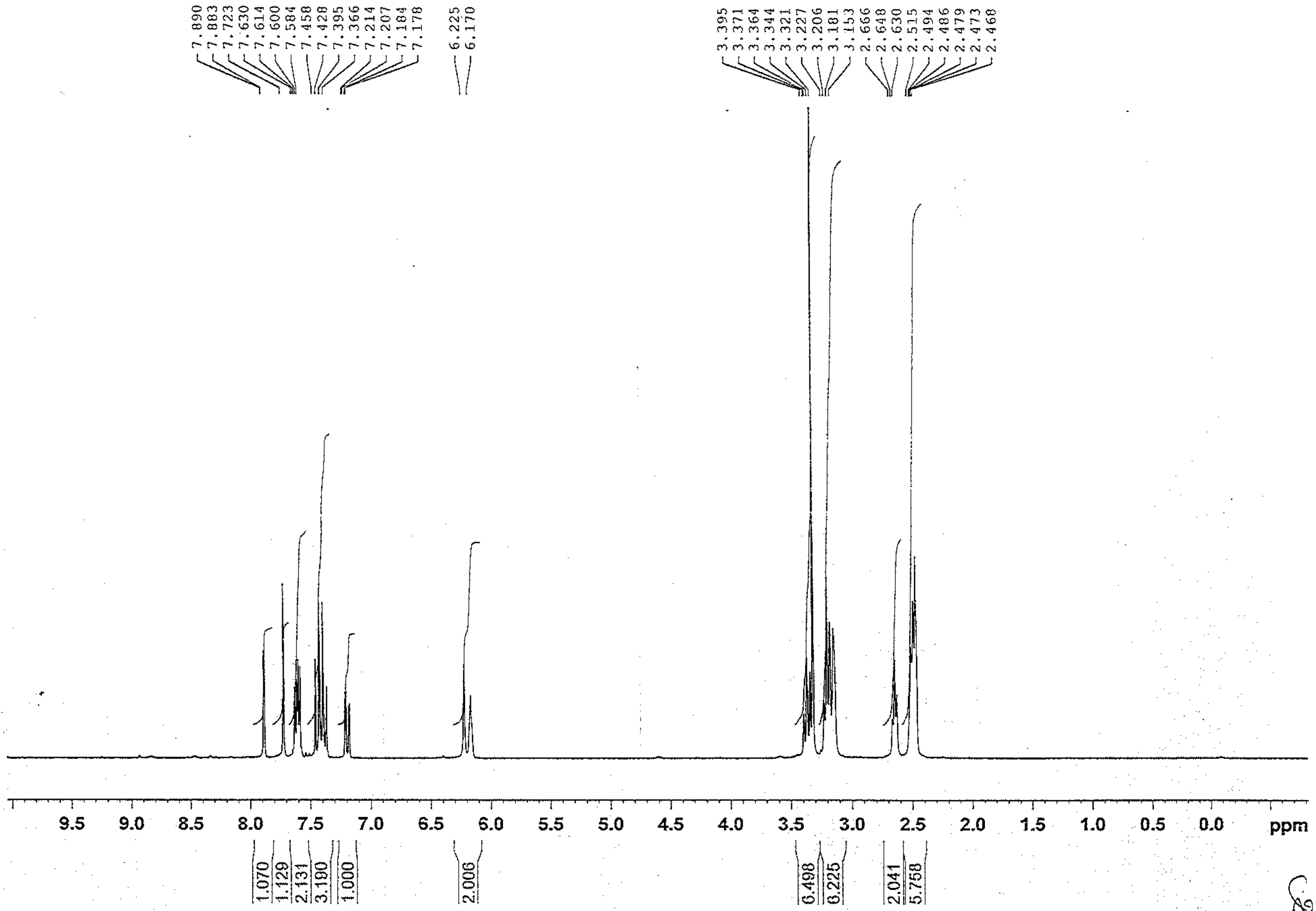

13C NMR of 0080/1847/031 in DMSO-d6
Date:04/04/09 AR.No:SE0409/01
{C:/LKT/aptuit Laurus/April 2009/11/1}

aptuit laurus

Anhydro 5

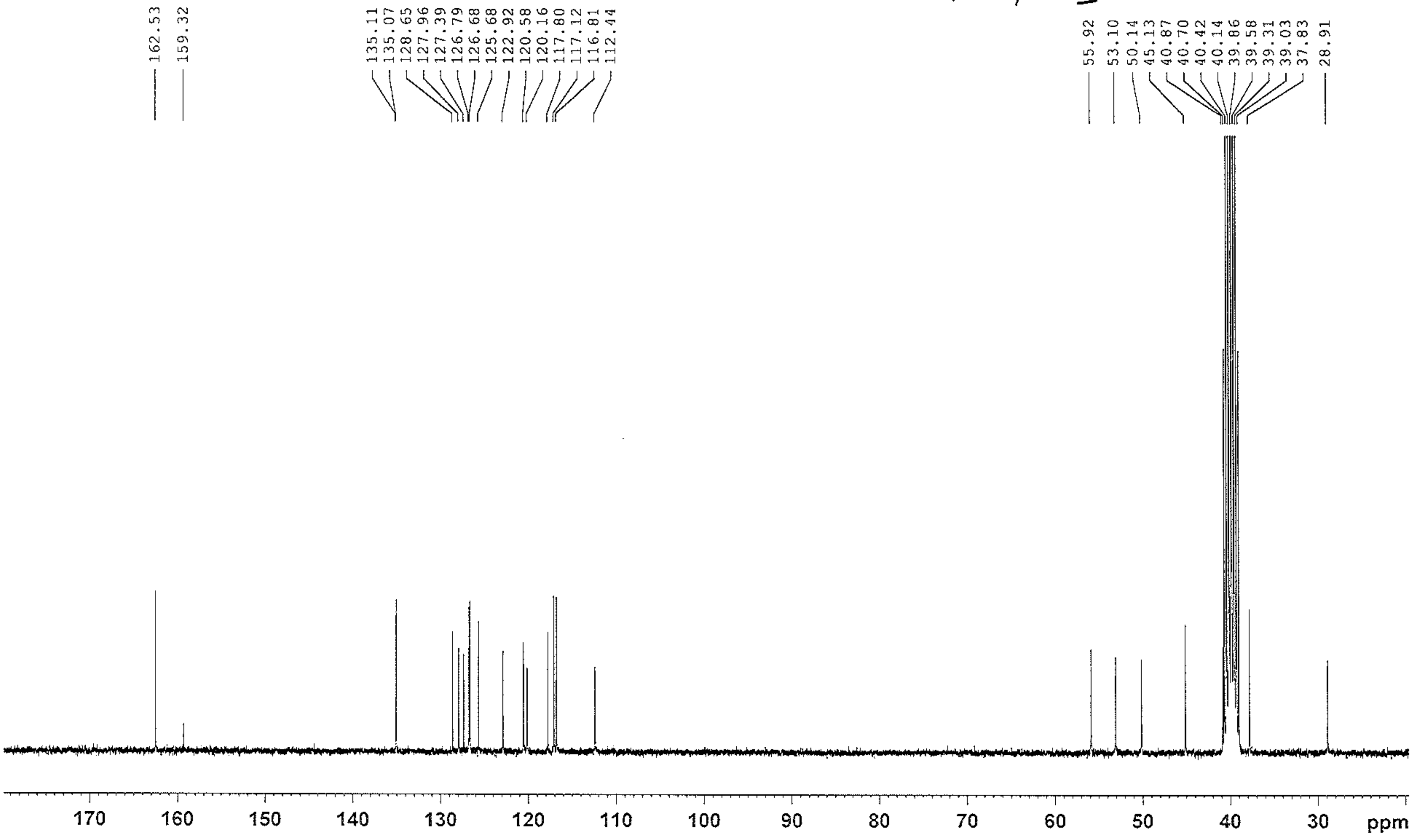

Analyst: Lakshmi Kumar.Tatini

aptuit laurus

Analytical Development

SAMPLE INFORMATION

Sample Name:	0080/ASt-imp/1847/031pre-1	Instrument Method Id 4820	
Sample Type:	Unknown	Acquired By:	Analyst
Vial:	56	Date Acquired:	7/31/2009 12:59:11 AM IST
Injection #:	1	Date Processed:	7/31/2009 9:40:50 AM IST
Injection Volume:	10.00 ul	Processing Method:	0080_RS
Sample Set Id 5596		Processing Method Id 5700	
Sample Set Name:	300709 01	Proc. Chnl. Descr.:	VWD AU On nm
Project Name: Sertindole_July_09		System Name LL_AD_LC_SYS011	
Result Id 5729	Result Set Id	Software: Empower 2 Software Build 2154	

Auto-Scaled Chromatogram

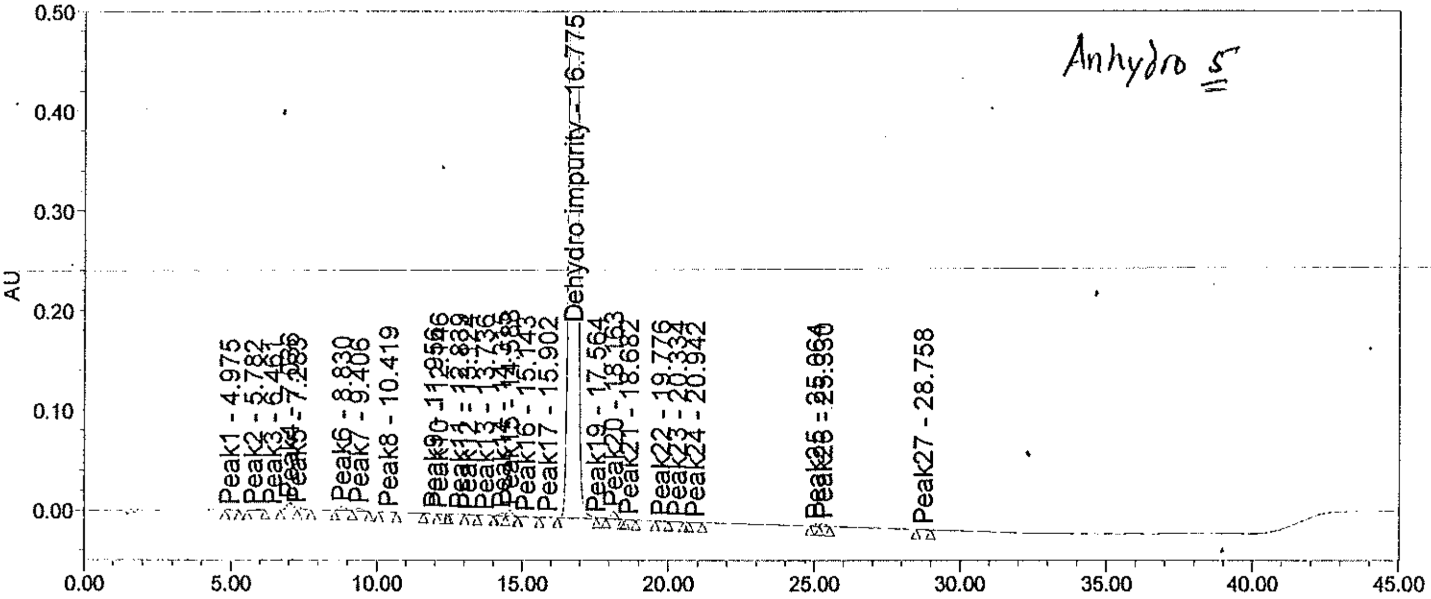

Peak Results

	Name	RT	Area (µV*sec)	% Area	RT Ratio	USP Resolution	USP Tailing	USP Plate Count
1	Peak1	4.97	4235.71	0.01	0.30		1.12	3159
2	Peak2	5.78	4677.11	0.02	0.34	2.14	1.09	2329
3	Peak3	6.46	3155.47	0.01	0.39	1.59	0.85	2132
4	Peak4	7.04	105617.05	0.37	0.42	1.35		4212
5	Peak5	7.28	25931.05	0.09	0.43			
6	Peak6	8.83	60238.89	0.21	0.53		1.18	7101
7	Peak7	9.41	36308.64	0.13	0.56	1.39	1.21	8564
8	Peak8	10.42	12944.27	0.05	0.62	2.16	0.84	7135
9	Peak9	11.96	12461.13	0.04	0.71			
10	Peak10	12.15	9117.20	0.03	0.72			
11	Peak11	12.84	15091.42	0.05	0.77			
12	Peak12	13.12	7469.25	0.03	0.78			

1H NMR of 0080/Ast-2/2198/121 in DMSO-d6
Date:- 31/07/09 ARNo :SE0709/024
{C:/LKT/Structure elucidation/July2009/59/1

Nor-Sertindole 9

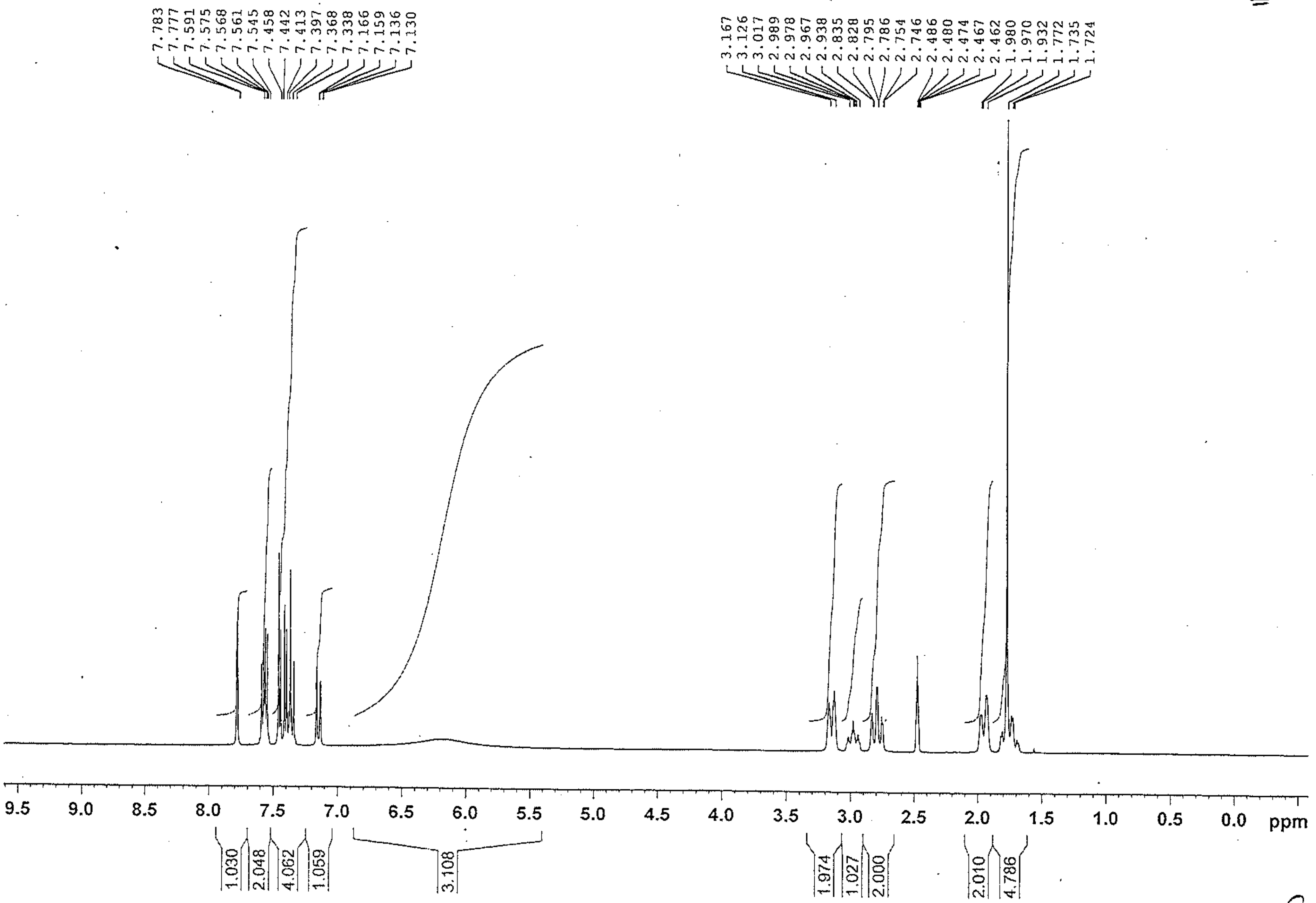

Analyst: Suresh Kumar.K

13C NMR of 0080/Ast-2/2198/121 in DMSO-d6
Date:- 31/07/09 ARNo :SE0709/024
{C:/LKT/Structure elucidation/July2009/62/1}

aptuit laurus

Nor -Sertindole 9

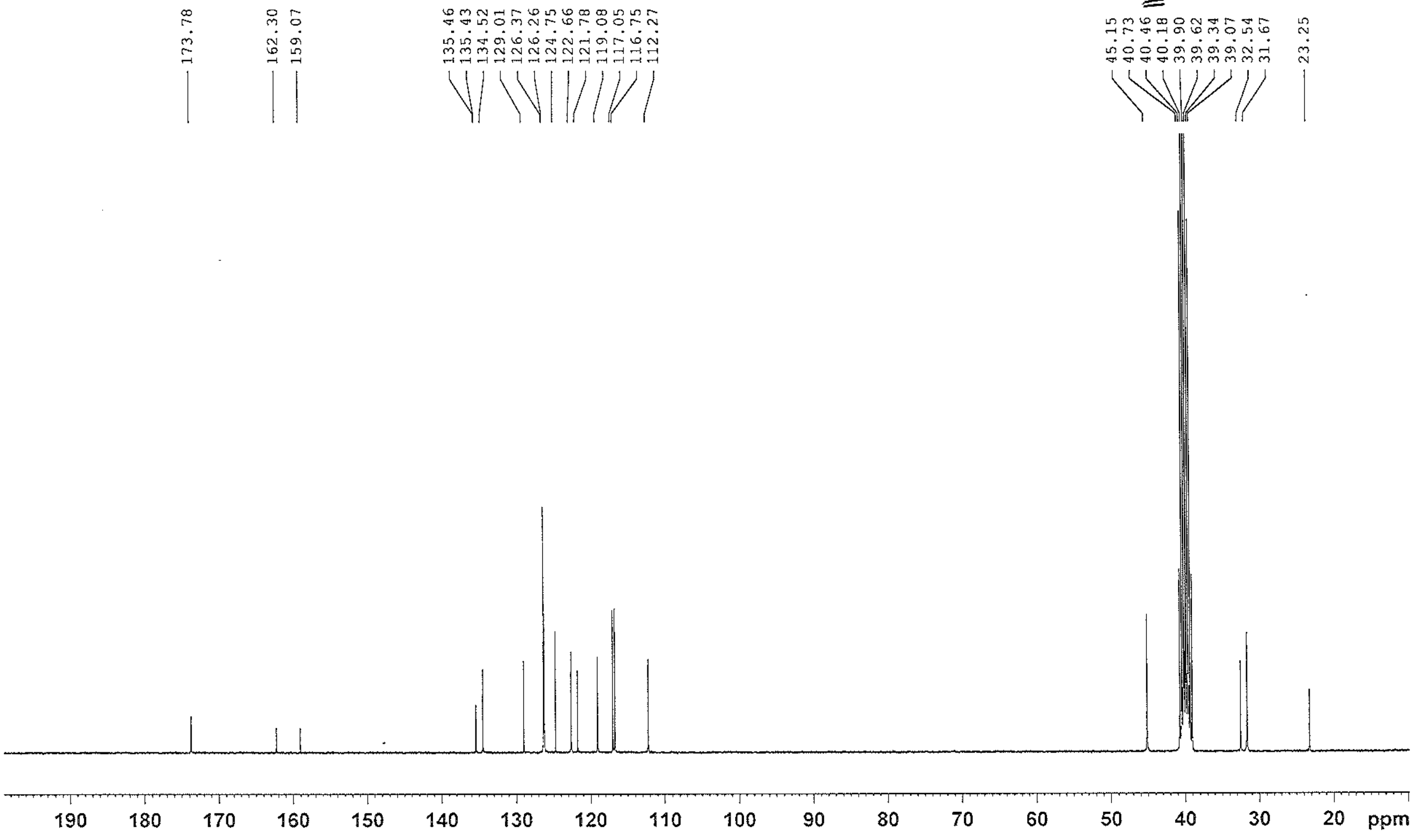

Analyst: Lakshmi Kumar.Tatini

aptuit laurus

Analytical Development

SAMPLE INFORMATION

Sample Name: 0080/A St-2/2198/121 pre-1
Sample Type: Unknown
Vial: 54
Injection #: 1
Injection Volume: 10.00 ul
Sample Set Id 5596
Sample Set Name: 300709 01
Project Name: Sertindole_July_09
Result Id 5694 Result Set Id

Instrument Method Id 4820
Acquired By: Analyst
Date Acquired: 7/30/2009 11:25:07 PM IST
Date Processed: 7/31/2009 9:37:05 AM IST
Processing Method: 0080_RS
Processing Method Id 5686
Proc. Chnl. Descr.: VWD AU On nm
System Name LL_AD_LC_SYS011
Software: Empower 2 Software Build 2154

Auto-Scaled Chromatogram

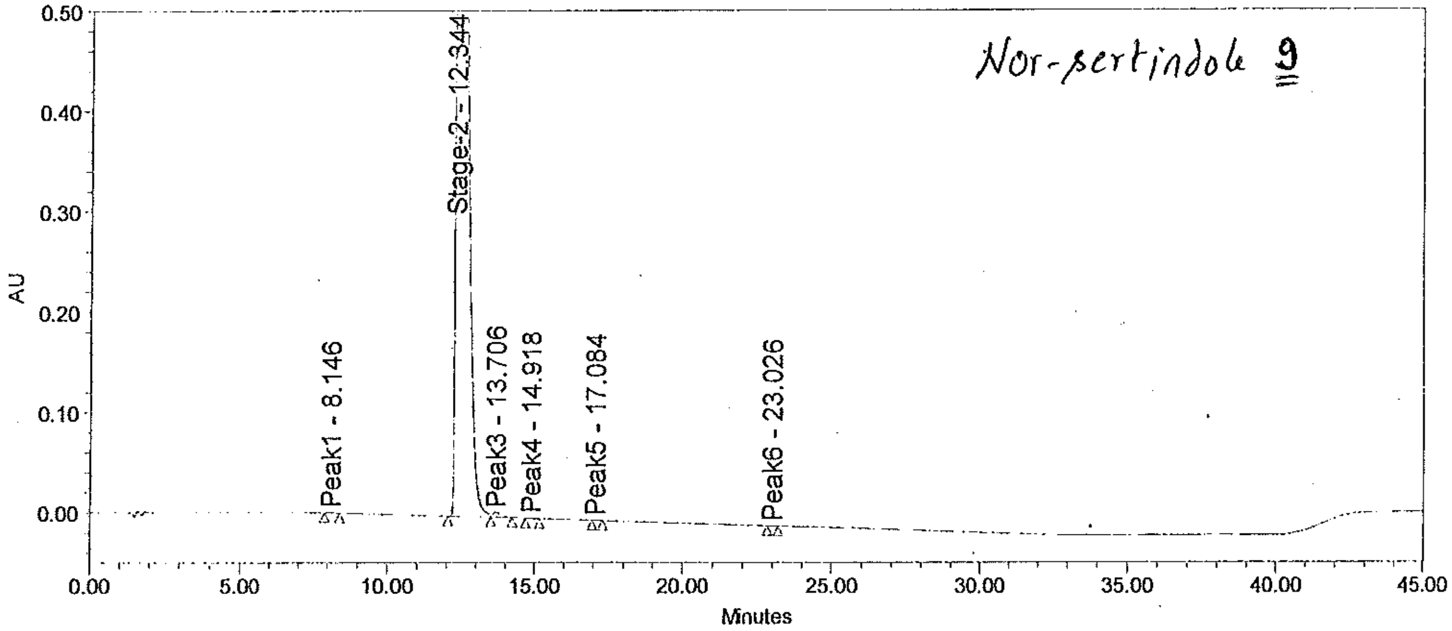

Peak Results

	Name	RT	Area (µV*sec)	% Area	RT Ratio	USP Resolution	USP Tailing	USP Plate Count
1	Peak1	8.15	6350.47	0.02	0.66		1.01	7002
2	Stage-2	12.34	28374700.77	99.61	1.00	8.07	3.15	6914
3	Peak3	13.71	94258.59	0.33	1.11			
4	Peak4	14.92	5302.13	0.02	1.21		1.04	30829
5	Peak5	17.08	2242.89	0.01	1.38	6.89	1.21	184174
6	Peak6	23.03	1774.47	0.01	1.87	19.19	0.86	75973

Signature / Date: [illegible] 31/07/09

1H NMR of 0080/2246/117 in DMSO-d6
Date:- 28/08/09 ARNO :SE0809/033
{C:/LKT/Structure elucidation/Aug-2009/72/1

1-(4-Bromophenyl) 21

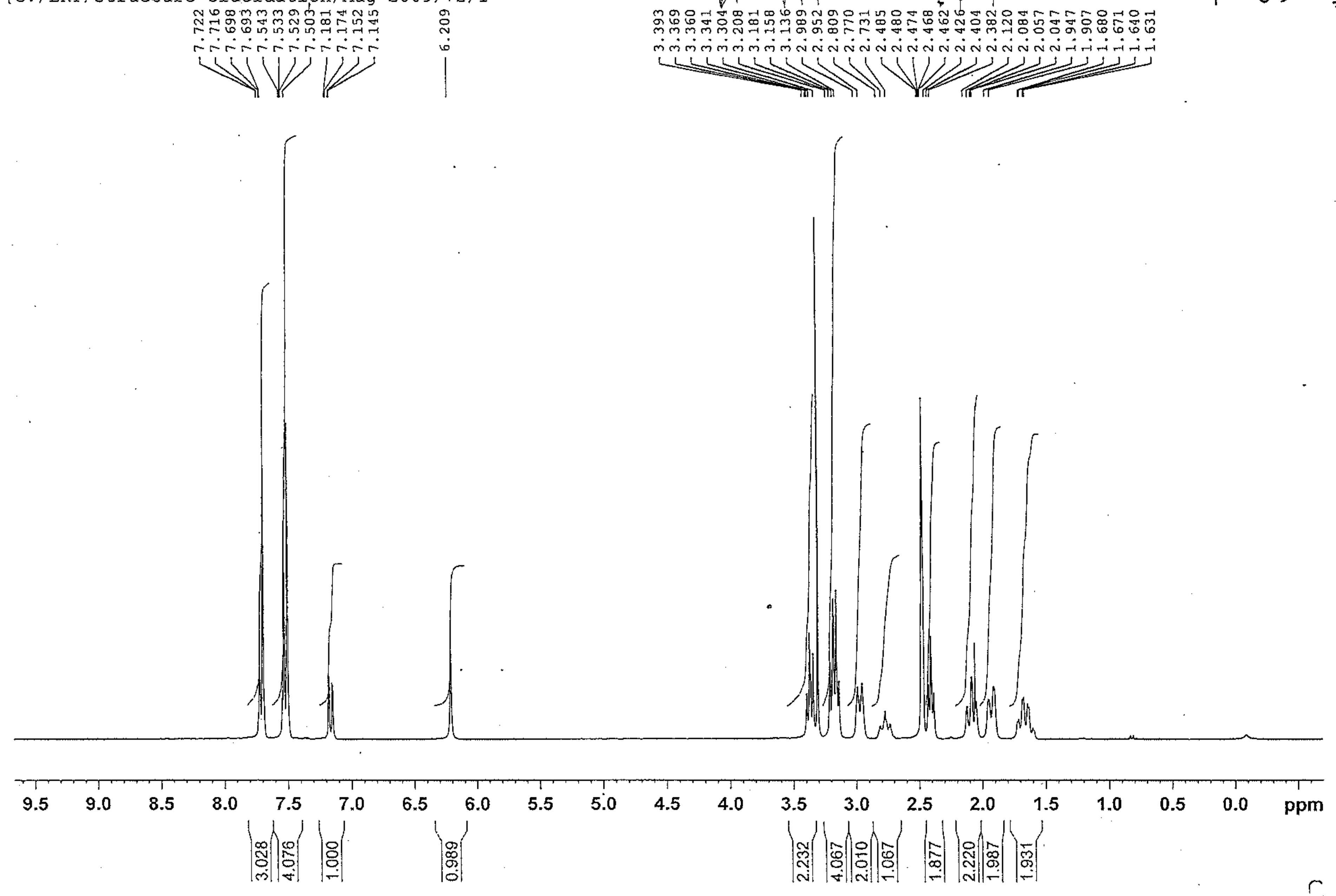

Analyst: Suresh Kumar.K

13C NMR of 0080/2246/117 in DMSO-d6
Date:- 28/08/09 ARNO :SE0809/033
{C:/LKT/Structure elucidation/Aug-2009/75/1}

aptuit laurus

1-(4-Bromophenyl) 21

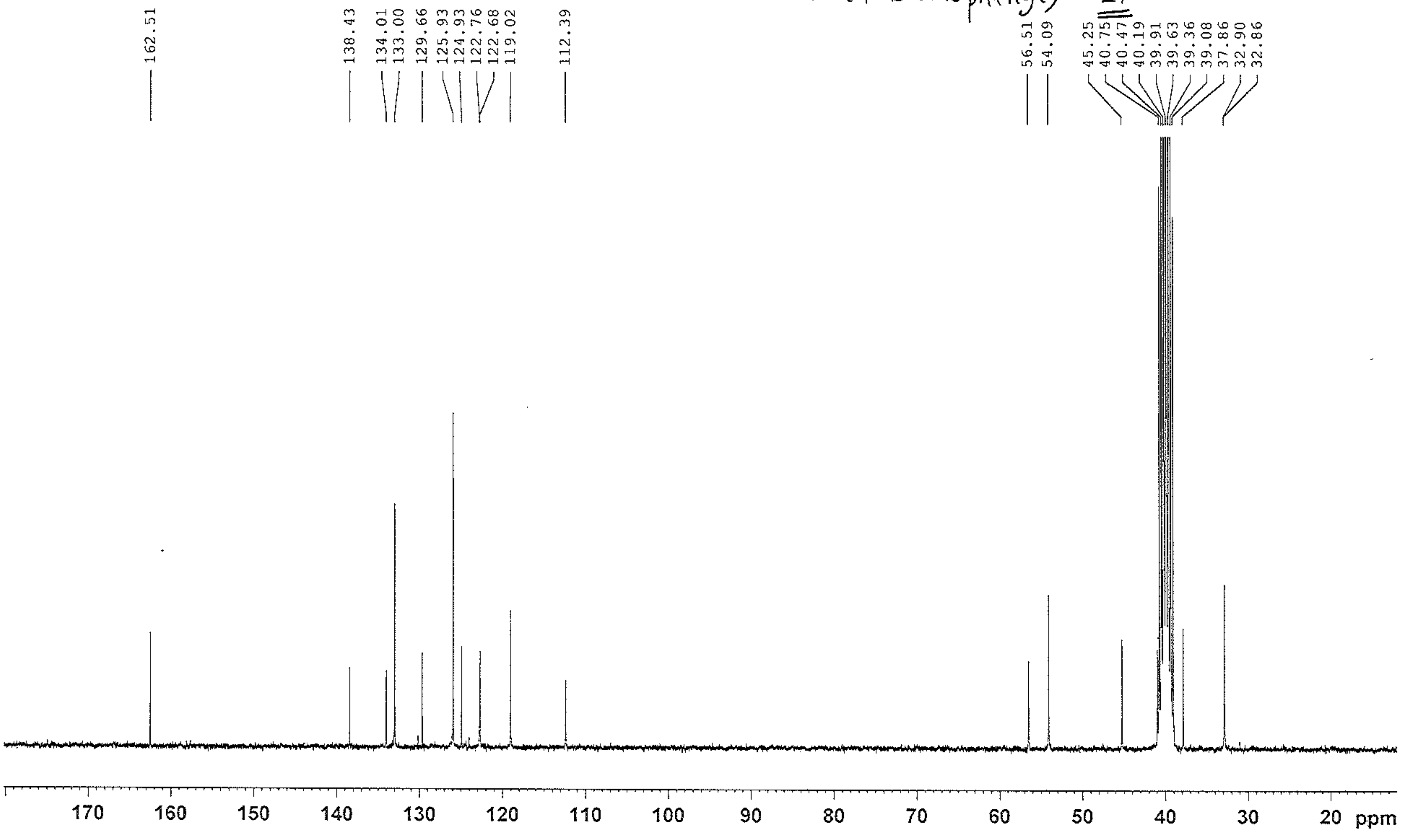

Analyst: Lakshmi Kumar.Tatini

.ıt laurus

Analytical Development

SAMPLE INFORMATION

Sample Name:	0080/2246/117(1-Bromo imp) Pre-1	Instrument Method Id 2893	
Sample Type:	Unknown	Acquired By:	Analyst
Vial:	25	Date Acquired:	9/8/2009 2:24:33 PM IST
Injection #:	1	Date Processed:	9/8/2009 5:05:41 PM IST
Injection Volume:	10.00 ul	Processing Method:	0080_Stage123_RS
Sample Set Id 11609		Processing Method Id 11621	
Sample Set Name:	080909_01	Proc. Chnl. Descr.:	VWD AU On nm
Project Name: Sertindole_July_09		System Name LL_AD_LC_SYS012	
Result Id 11635	Result Set Id	Software: Empower 2 Software Build 2154	

Auto-Scaled Chromatogram

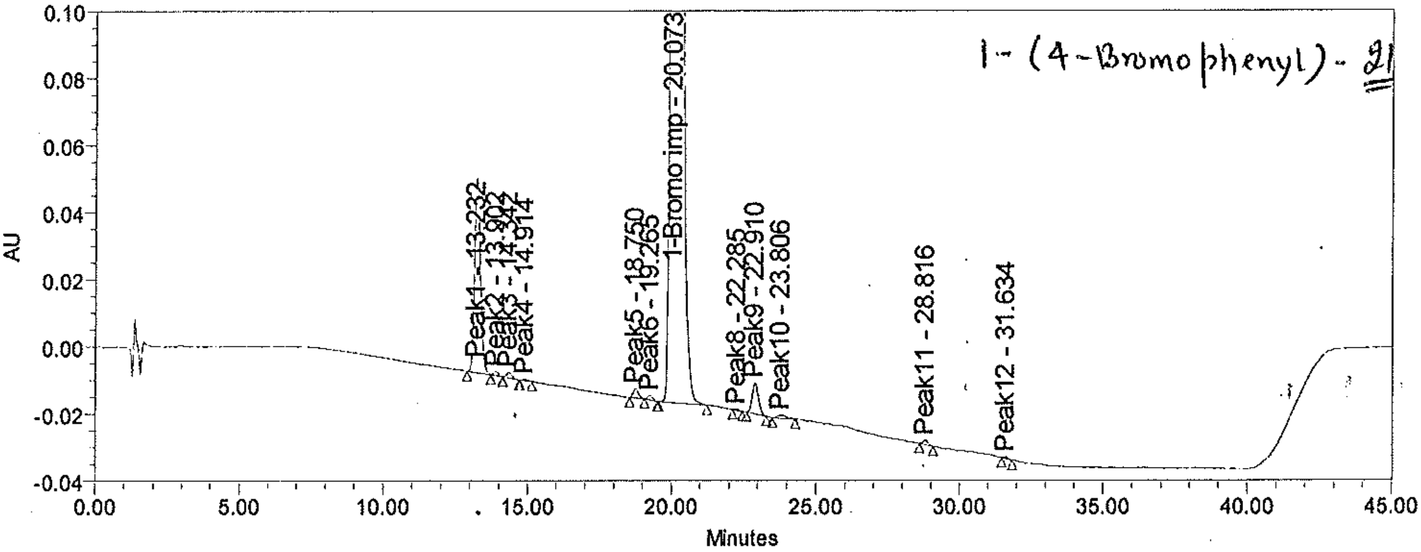

Peak Results

	Name	RT	Area (µV*sec)	% Area	RT Ratio	USP Tailing	USP Plate Count	USP Resolution
1	Peak1	13.23	805021.53	2.83	0.659	1.23	21240	
2	Peak2	13.90	12461.86	0.04	0.693	1.15	34128	1.95
3	Peak3	14.34	20947.52	0.07	0.714	1.45	26039	1.32
4	Peak4	14.91	4943.95	0.02	0.743	1.39	42461	1.77
5	Peak5	18.75	36363.78	0.13	0.934	1.24	44034	11.81
6	Peak6	19.27	14998.50	0.05	0.960	1.23	54606	1.50
7	1-Bromo imp	20.07	27356173.03	96.19	1.000	1.39	32044	2.06
8	Peak8	22.29	3448.03	0.01	1.110	1.00	82094	5.82
9	Peak9	22.91	138750.59	0.49	1.141	1.17	51773	1.74
10	Peak10	23.81	22591.38	0.08	1.186	1.40	26437	1.64
11	Peak11	28.82	17632.31	0.06	1.436	1.10	112881	9.62
12	Peak12	31.63	5890.80	0.02	1.576	1.18	205970	8.69

1H NMR of 0080/2198/177 in DMSO-d6
Date:- 05/08/09 ARNO :SE0809/05
{C:/LKT/Structure elucidation/Aug-2009/16/1

5-Bromo 27

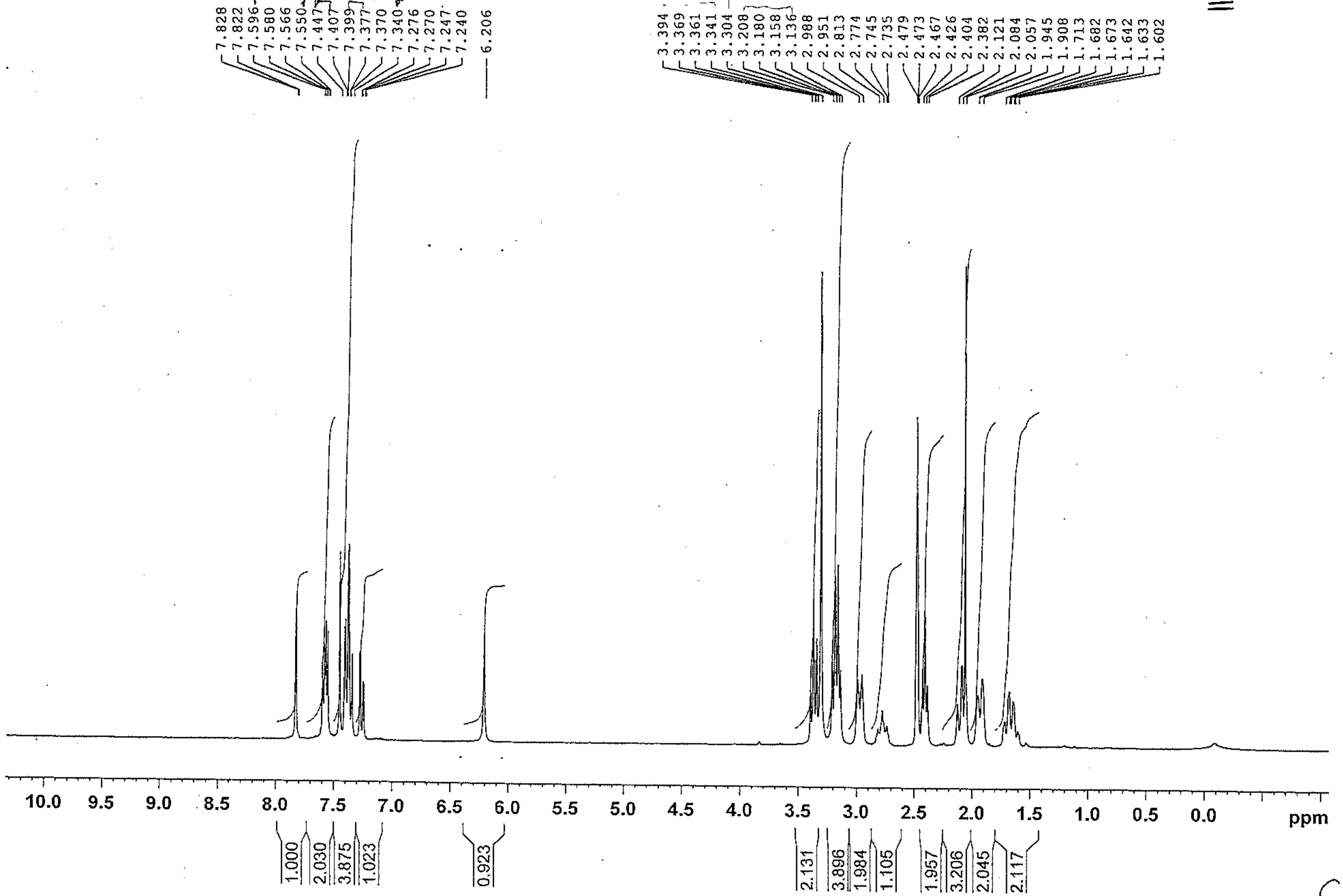

Analyst: Suresh Kumar.K

13C NMR of 0080/2198/177 in DMSO-d6
Date:- 05/08/09 ARNO :SE0809/05
{C:/LKT/Structure elucidation/Aug-2009/19/1}

aptuit laurus

5-Bromo 27

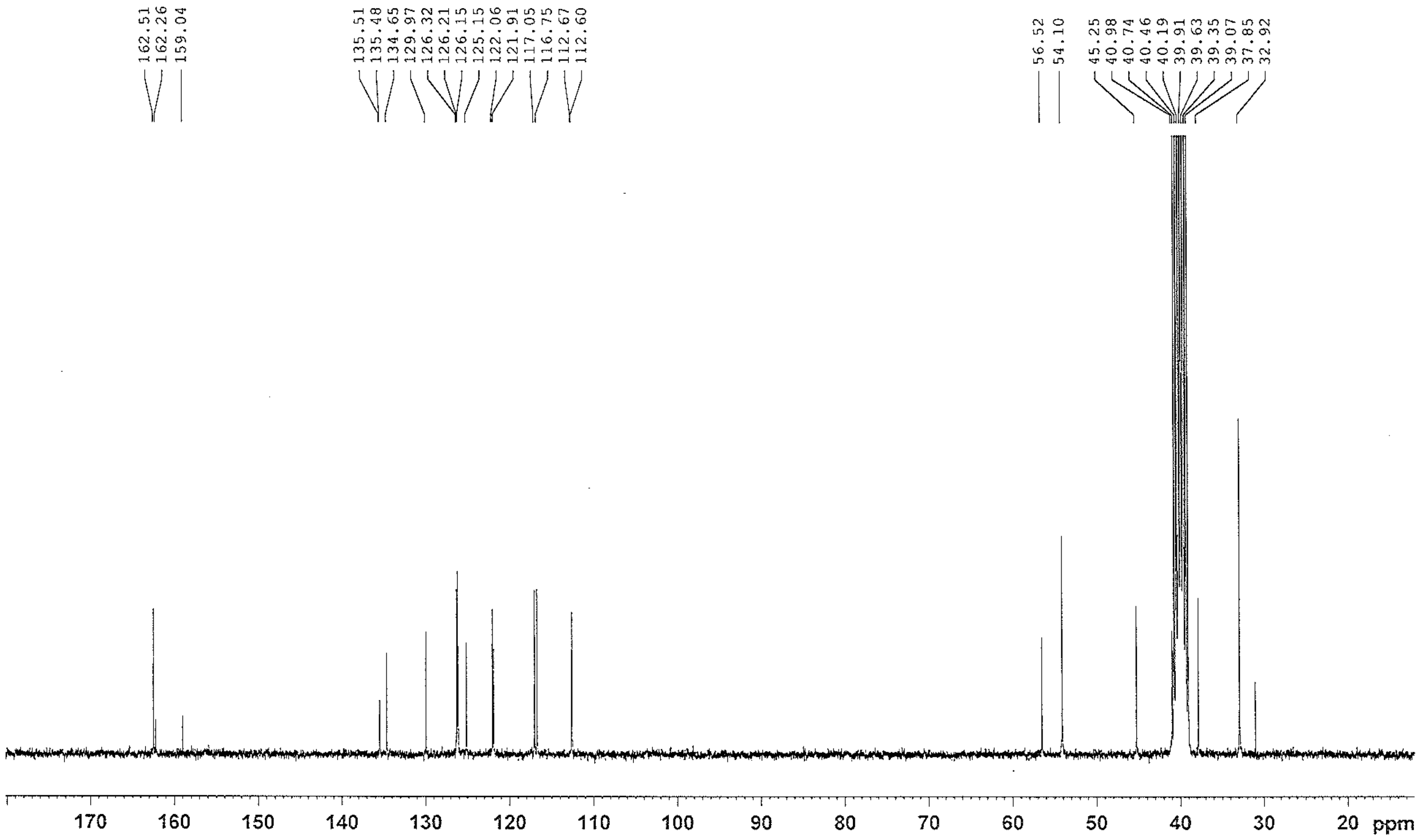

Analyst: Lakshmi Kumar.Tatini

aptuit laurus

Analytical Development

SAMPLE INFORMATION

Sample Name: 0080/2198/177(5-Bromo)Pre-1
Sample Type: Unknown
Vial: 57
Injection #: 1
Injection Volume: 10.00 ul
Sample Set Id 6733
Sample Set Name: 040809_01
Project Name: Sertindole_July_09
Result Id 6994 Result Set Id

Instrument Method Id 6729
Acquired By: Analyst
Date Acquired: 8/5/2009 9:00:15 AM IST
Date Processed: 8/5/2009 11:52:43 AM IST
Processing Method: 0080_Stage123_RS
Processing Method Id 6978
Proc. Chnl. Descr.:
System Name LL_AD_LC_SYS006
Software: Empower 2 Software Build 2154

Auto-Scaled Chromatogram

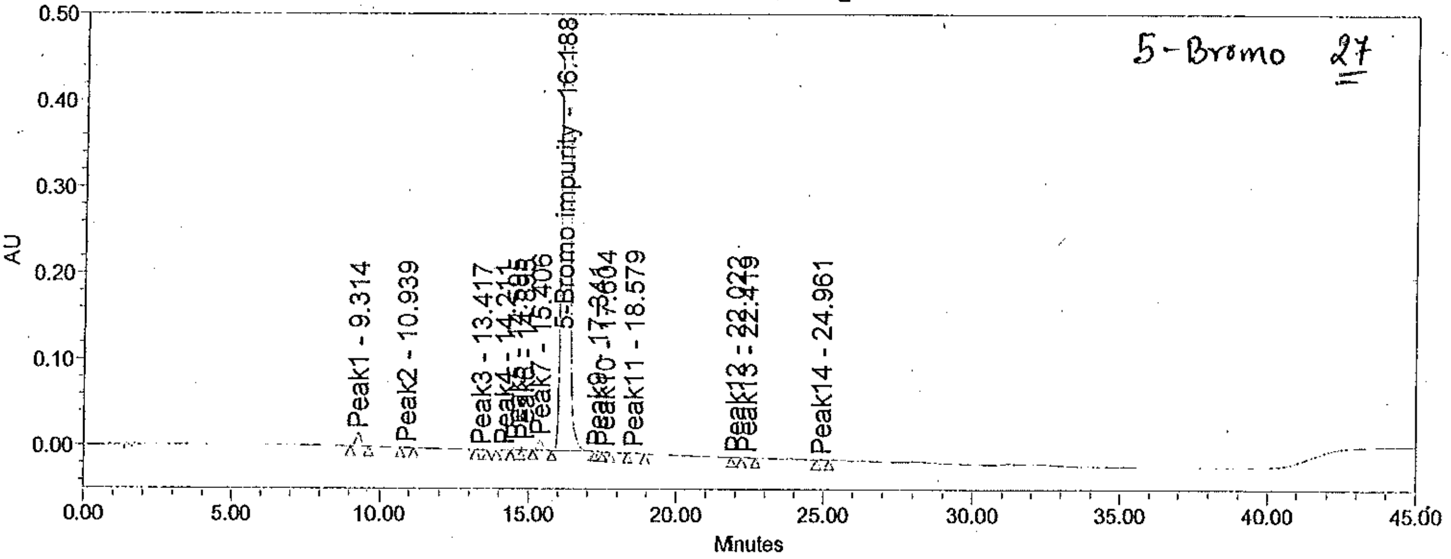

Peak Results

	Name	RT	Area (µV*sec)	%Area	RT Ratio	USP Resolution	USP Tailing	USP Plate Count
1	Peak1	9.31	169853.55	0.82	0.58		1.11	15386
2	Peak2	10.94	6288.65	0.03	0.68	5.10	1.36	18075
3	Peak3	13.42	1289.42	0.01	0.83	7.63	0.93	29975
4	Peak4	14.21	9390.67	0.05	0.88	2.58	0.94	37378
5	Peak5	14.60	68669.33	0.33	0.90			25965
6	Peak6	14.89	71981.76	0.35	0.92			19277
7	Peak7	15.41	140468.91	0.68	0.95		1.34	39381
8	5-Bromo impurity	16.19	20124412.85	97.52	1.00	2.29	1.25	32573
9	Peak9	17.34	2364.73	0.01	1.07	4.03	1.26	103152
10	Peak10	17.60	2355.05	0.01	1.09	1.15	1.24	76840
11	Peak11	18.58	25197.58	0.12	1.15	3.09	1.44	40669
12	Peak12	22.02	3613.92	0.02	1.36	10.36	1.18	96388
13	Peak13	22.42	6470.13	0.03	1.38	1.27	1.36	68778
14	Peak14	24.96	3719.33	0.02	1.54	7.97	1.33	114360

Signature / Date: [illegible] 05/08/09

1H NMR of 0080/2154/200 in DMSO-d6
Date:- 29/07/09 ARNo :SE0709/022
{C:/LKT/Structure elucidation/July2009/54/1

Bis-alkylated 28

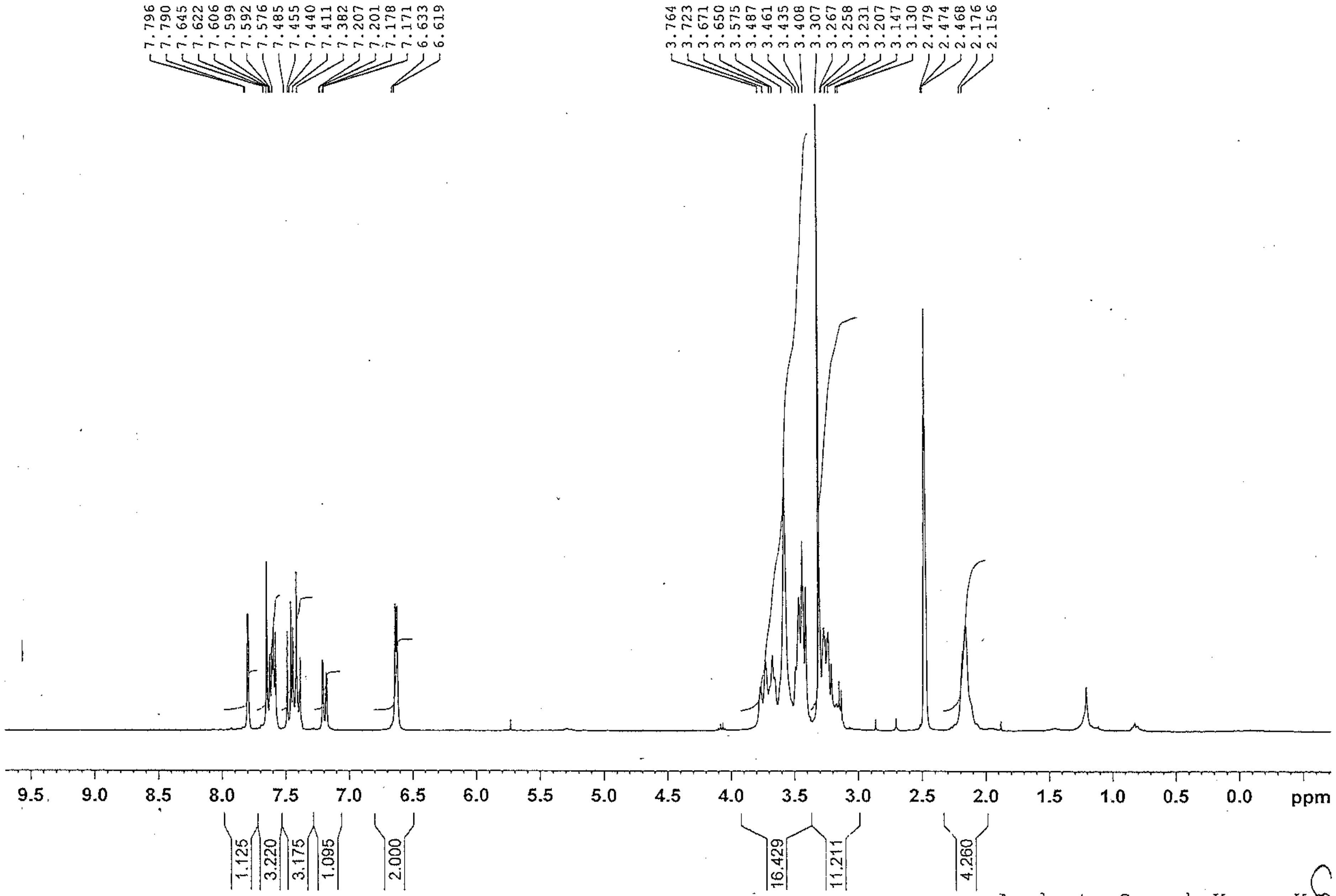

Analyst: Suresh Kumar.K

13C NMR of 0080/2154/200 in DMSO-d6
Date:- 29/07/09 ARNo :SE0709/022
{C:/LKT/Structure elucidation/July2009/57/1}

aptuit laurus

Bis-alkylated 28

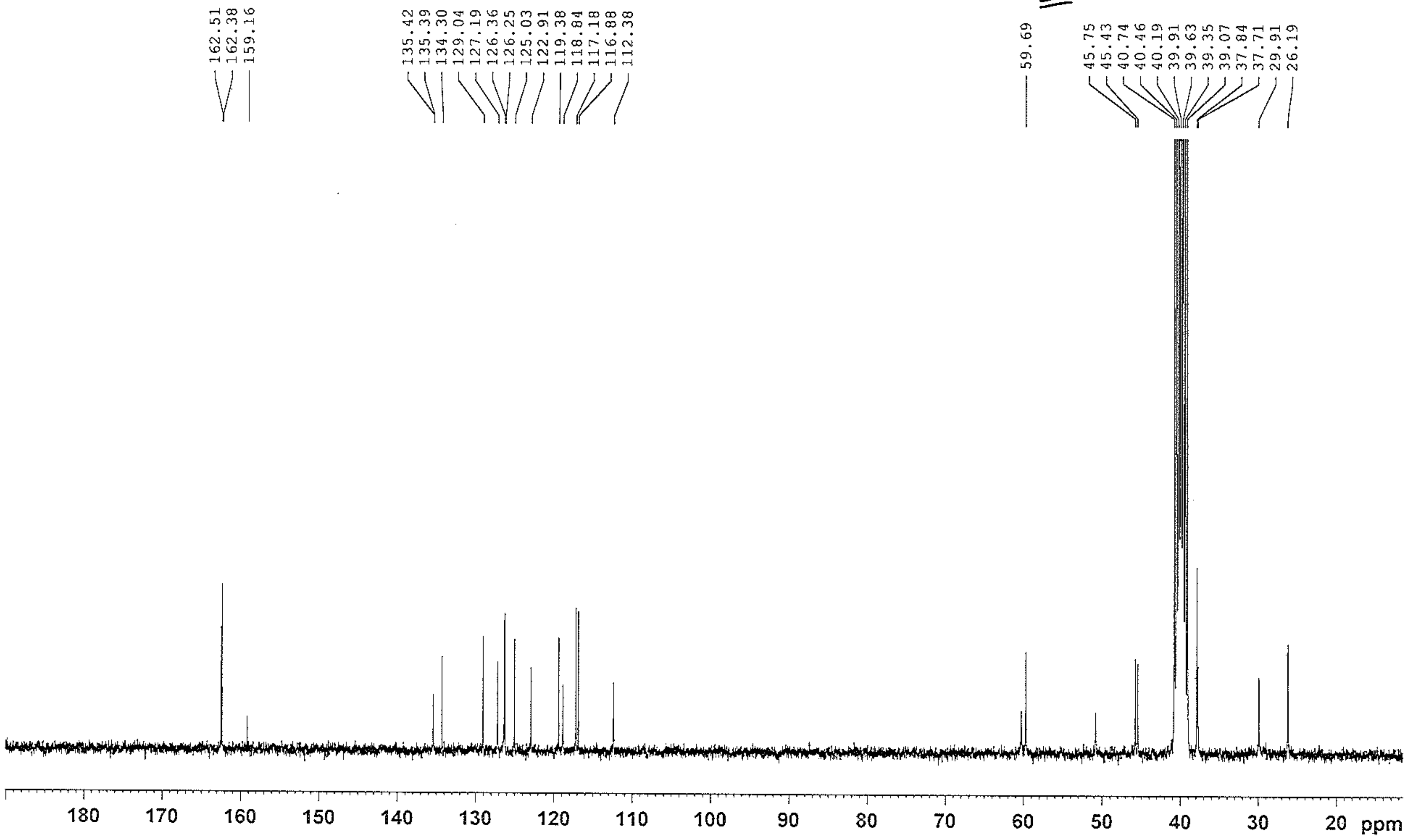

Analyst: Lakshmi Kumar.Tatini

aptuit laurus

Analytical Development

SAMPLE INFORMATION

Sample Name:	0080/2154/200(~~dimer~~ Bis imp) pre-1	Instrument Method Id 4820	
Sample Type:	Unknown	Acquired By:	Analyst
Vial:	62	Date Acquired:	7/31/2009 5:41:41 AM IST
Injection #:	1	Date Processed:	7/31/2009 10:00:23 AM IST
Injection Volume:	10.00 ul	Processing Method:	0080_RS
Sample Set Id 5596		Processing Method Id 5767	
Sample Set Name:	300709 01	Proc. Chnl. Descr.:	VWD AU On nm
Project Name: Sertindole_July_09		System Name LL_AD_LC_SYS011	
Result Id 5791	Result Set Id	Software: Empower 2 Software Build 2154	

31/07/09

Auto-Scaled Chromatogram

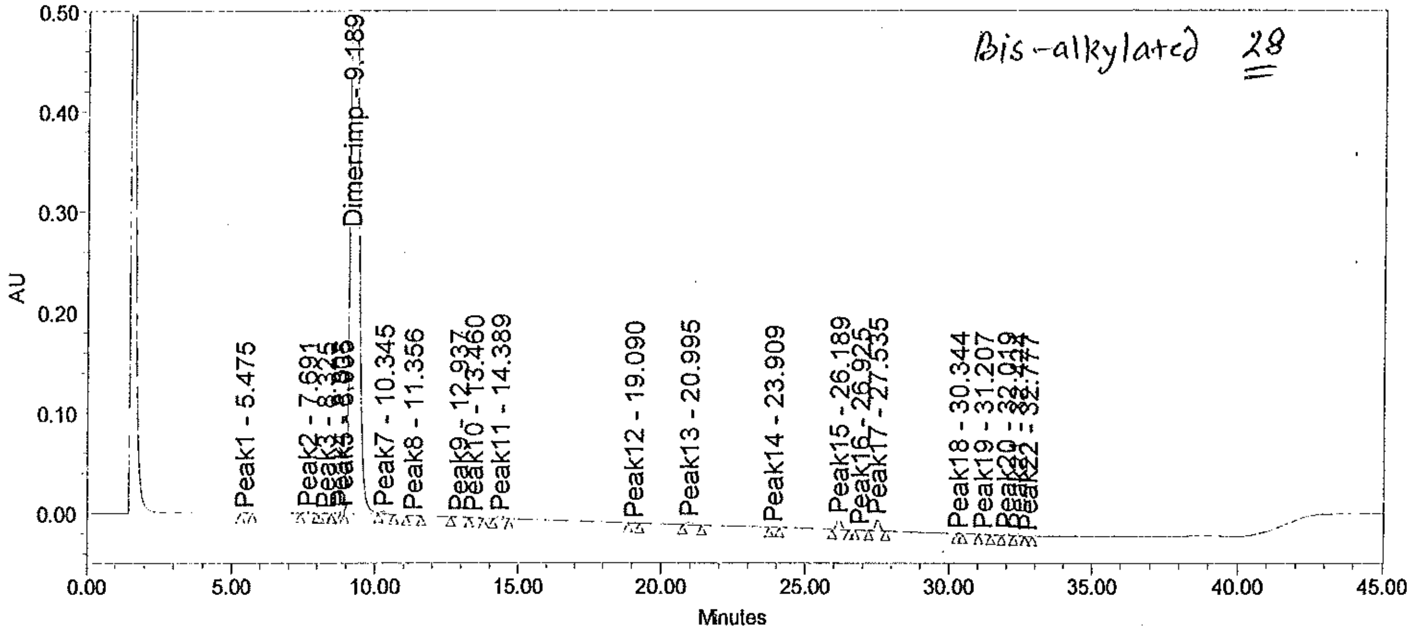

Peak Results

	Name	RT	Area (µV*sec)	% Area	RT Ratio	USP Resolution	USP Tailing	USP Plate Count
1	Peak1	5.47	2896.68	0.02	0.60		1.23	3663
2	Peak2	7.69	14796.54	0.10	0.84	5.94	1.10	7305
3	Peak3	8.33	2672.10	0.02	0.91	1.83	0.84	10900
4	Peak4	8.81	9477.23	0.06	0.96			
5	Peak5	8.94	14247.40	0.10	0.97			
6	Dimer imp	9.19	14262222.61	96.67	1.00		1.90	6663
7	Peak7	10.35	32588.51	0.22	1.13	2.81	1.28	13121
8	Peak8	11.36	7449.18	0.05	1.24	2.80	0.93	13919
9	Peak9	12.94	19684.40	0.13	1.41	3.63	1.19	9482
10	Peak10	13.46	12459.01	0.08	1.46	1.17	1.46	22709
11	Peak11	14.39	33203.96	0.23	1.57	2.55	1.10	29855
12	Peak12	19.09	1939.73	0.01	2.08	13.90	0.84	63712

1H NMR of 0080/2198/090 in DMSO-d6
Date:- 29/07/09 ARNo :SE0709/023
{C:/LKT/Structure elucidation/July2009/51/1

N-oxide 29

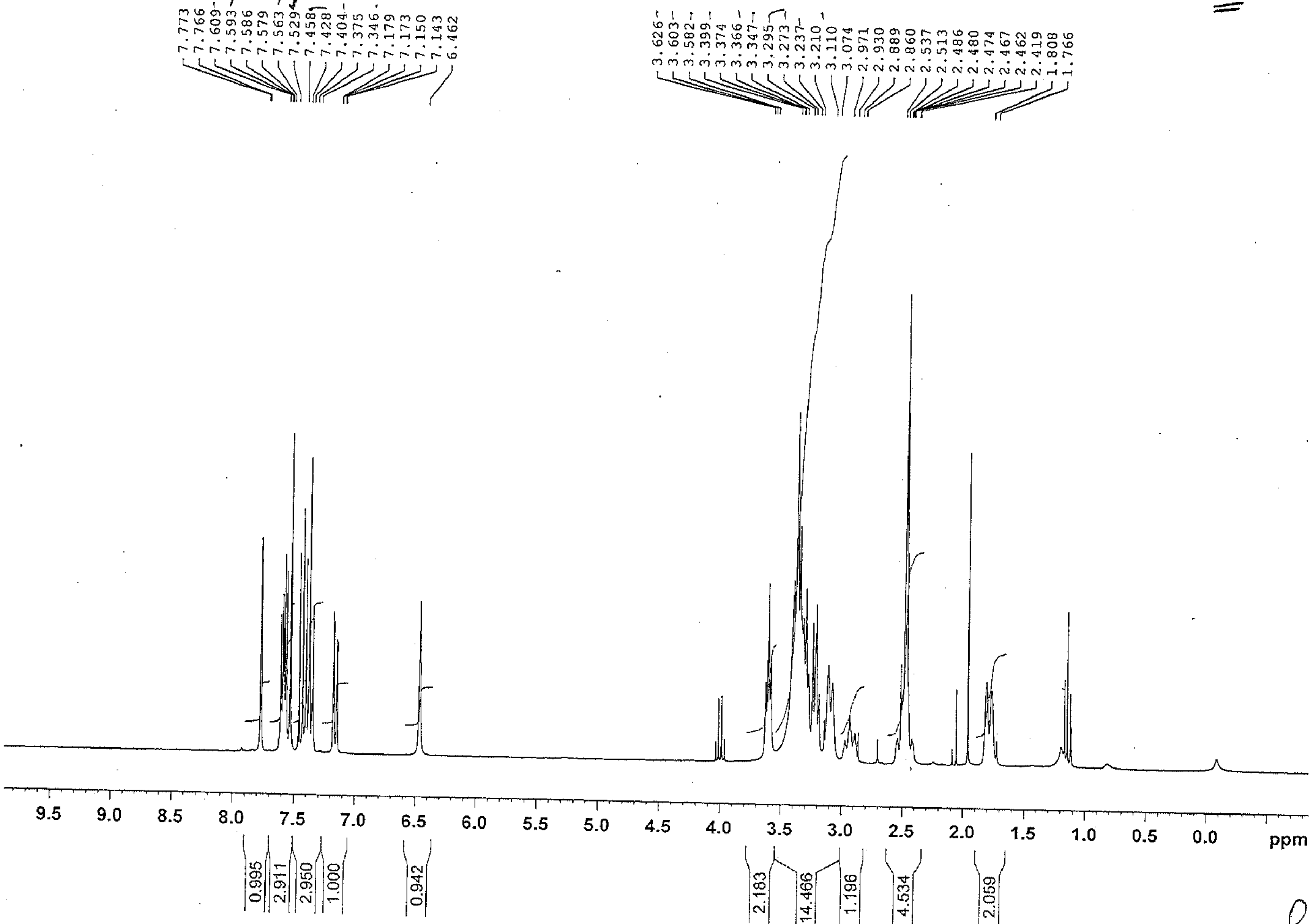

Analyst: Suresh Kumar.K

13C NMR of 0080/2198/090 in DMSO-d6
Date:- 30/07/09 ARNo :SE0709/023
{C:/LKT/Structure elucidation/July2009/58/1}

aptuit laurus

N-oxide 29

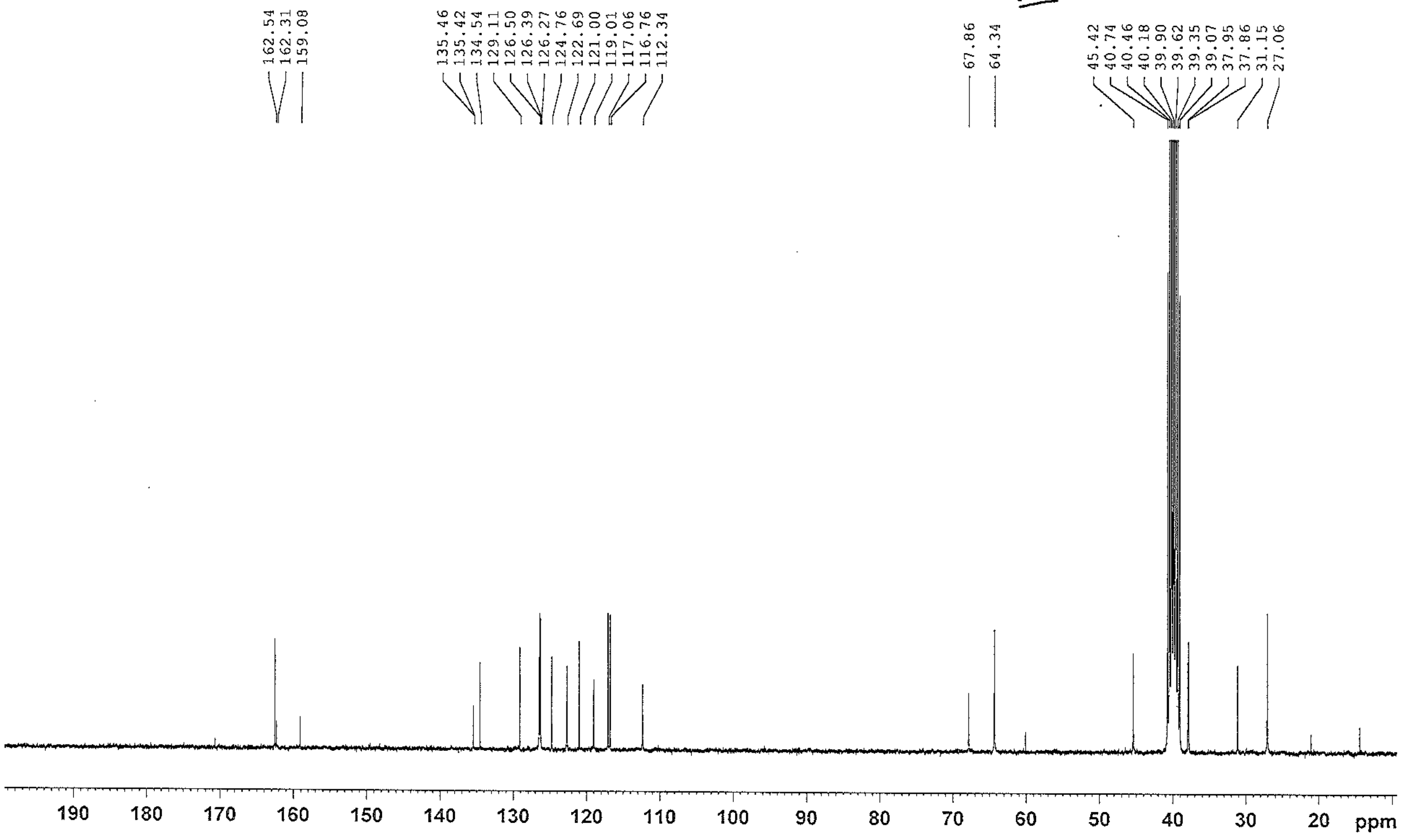

Analyst: Lakshmi Kumar.Tatini

aptuit laurus

Analytical Development

SAMPLE INFORMATION

Sample Name:	0080/2198/090(N-Oxide)pre-1	Instrument Method Id 4820	
Sample Type:	Unknown	Acquired By:	Analyst
Vial:	60	Date Acquired:	7/31/2009 4:07:35 AM IST
Injection #:	1	Date Processed:	7/31/2009 9:53:06 AM IST
Injection Volume:	10.00 ul	Processing Method:	0080_RS
Sample Set Id 5596		Processing Method Id 5748	
Sample Set Name:	300709 01	Proc. Chnl. Descr.:	VWD AU On nm
Project Name: Sertindole_July_09		System Name LL_AD_LC_SYS011	
Result Id 5762	Result Set Id	Software: Empower 2 Software Build 2154	

Auto-Scaled Chromatogram

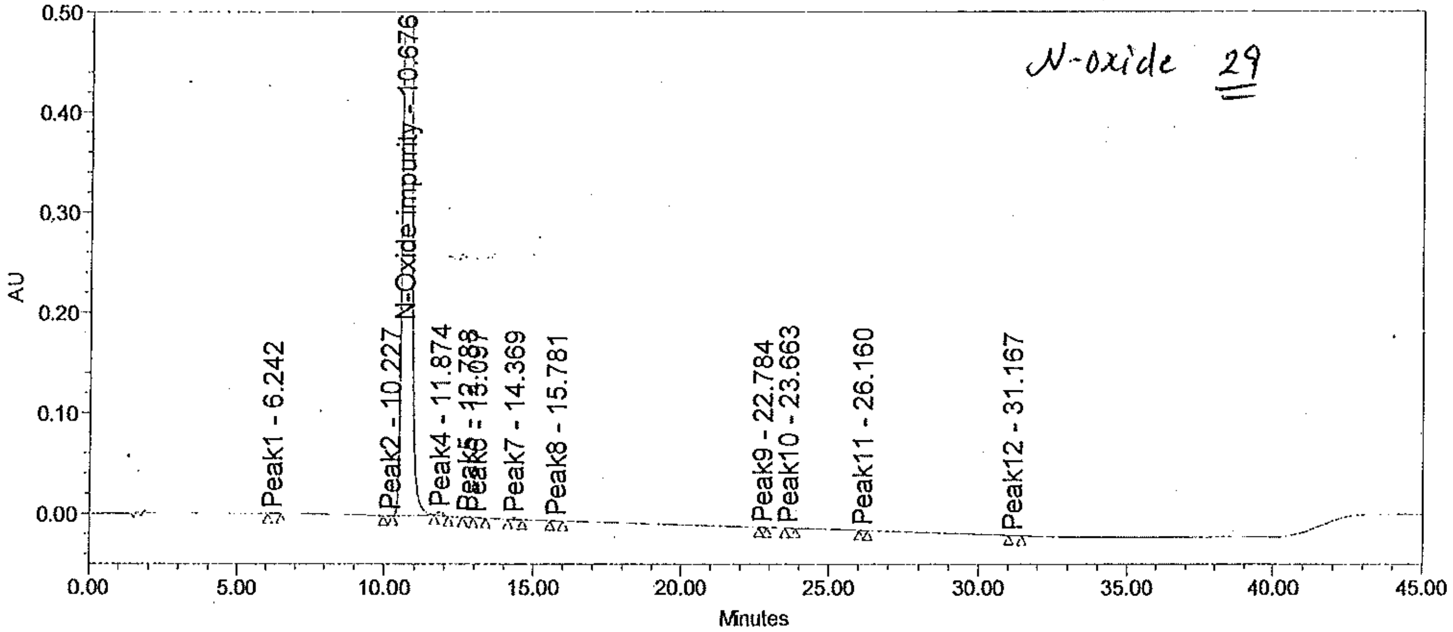

Peak Results

	Name	RT	Area (µV*sec)	% Area	RT Ratio	USP Resolution	USP Tailing	USP Plate Count
1	Peak1	6.24	3592.17	0.02	0.58		1.09	4024
2	Peak2	10.23	5303.52	0.02	0.96			
3	N-Oxide impurity	10.68	23008159.93	99.51	1.00		1.26	12833
4	Peak4	11.87	44388.07	0.19	1.11	3.24	1.19	18072
5	Peak5	12.79	14219.19	0.06	1.20	2.51		20467
6	Peak6	13.10	8240.95	0.04	1.23			
7	Peak7	14.37	24744.88	0.11	1.35		1.11	27527
8	Peak8	15.78	3249.25	0.01	1.48	4.54	0.98	29353
9	Peak9	22.78	1289.54	0.01	2.13	27.23	0.91	86436
10	Peak10	23.66	3557.38	0.02	2.22	3.38	1.12	170517
11	Peak11	26.16	1693.83	0.01	2.45	8.43	1.06	189542
12	Peak12	31.17	3847.68	0.02	2.92	14.84	1.41	169601

SampleName 0080/2198/090(N-Oxide)pre-1

Date Acquired 7/31/2009 4:07:35 AM IST

Signature / Date: Rao 31/07/09

aptuit laurus

Analytical Development

SAMPLE INFORMATION

Sample Name:	0.1%(Solution)Accuracy	Instrument Method Id 12617	
Sample Type:	Unknown	Acquired By:	Analyst
Vial:	60	Date Acquired:	9/17/2009 6:13:52 AM IST
Injection #:	1	Date Processed:	9/17/2009 9:36:25 AM IST
Injection Volume:	10.00 ul	Processing Method:	0080 _Stage 4 _RS
Sample Set Id 12704		Processing Method Id 12819	
Sample Set Name:	160909 02	Proc. Chnl. Descr.:	VWD AU On nm
Project Name: Sertindole_July_09		System Name LL_AD_LC_SYS011	
Result Id 12832	Result Set Id	Software: Empower 2 Software Build 2154	

Auto-Scaled Chromatogram

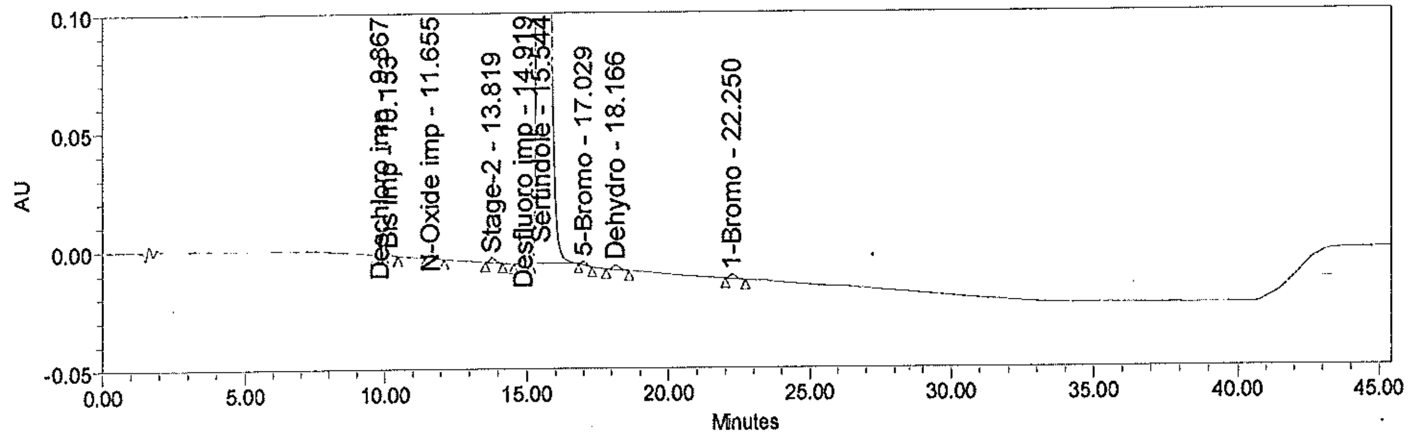

Peak Results

	Name	RT	Area (µV*sec)	%Area	RT Ratio	USP Resolution	USP Tailing	USP Plate Count	s/n
1	Deschloro imp	9.87	12966.78	0.05	0.63				21.62
2	Bis imp	10.15	14725.13	0.05	0.65				21.67
3	N-Oxide imp	11.66	22585.48	0.08	0.75		1.54	11568	31.81
4	Stage-2	13.82	29223.62	0.11	0.89	5.12	1.26	20203	46.26
5	Desfluoro imp	14.92	32837.84	0.12	0.96	2.50	0.84	16831	45.99
6	Sertindole	15.54	26807667.48	99.30		1.31	1.40	17973	34756.09
7	5-Bromo	17.03	19482.56	0.07	1.10	3.48	1.31	32746	32.30
8	Dehydro	18.17	30939.38	0.11	1.17	2.71	1.41	27197	42.44
9	1-Bromo	22.25	27398.08	0.10	1.43	9.17	1.39	44192	39.70

Base line noise : 0.044

aptuit laurus

Analytical Development

SAMPLE INFORMATION

Sample Name:	0080 sst solution	Instrument Method Id 1302	
Sample Type:	Unknown	Acquired By:	Analyst
Vial:	14	Date Acquired:	10/24/2009 1:37:43 PM IST
Injection #:	1	Date Processed:	10/25/2009 9:03:50 AM IST
Injection Volume:	10.00 ul	Processing Method:	0080 _Stage4 _RS
Sample Set Id 2253		Processing Method Id 2319	
Sample Set Name:	241009 01	Proc. Chnl. Descr.:	VWD AU On nm
Project Name: Sertindole_OCT_09		System Name LL_AD_LC_SYS012	
Result Id 2330	Result Set Id	Software: Empower 2 Software Build 2154	

Auto-Scaled Chromatogram

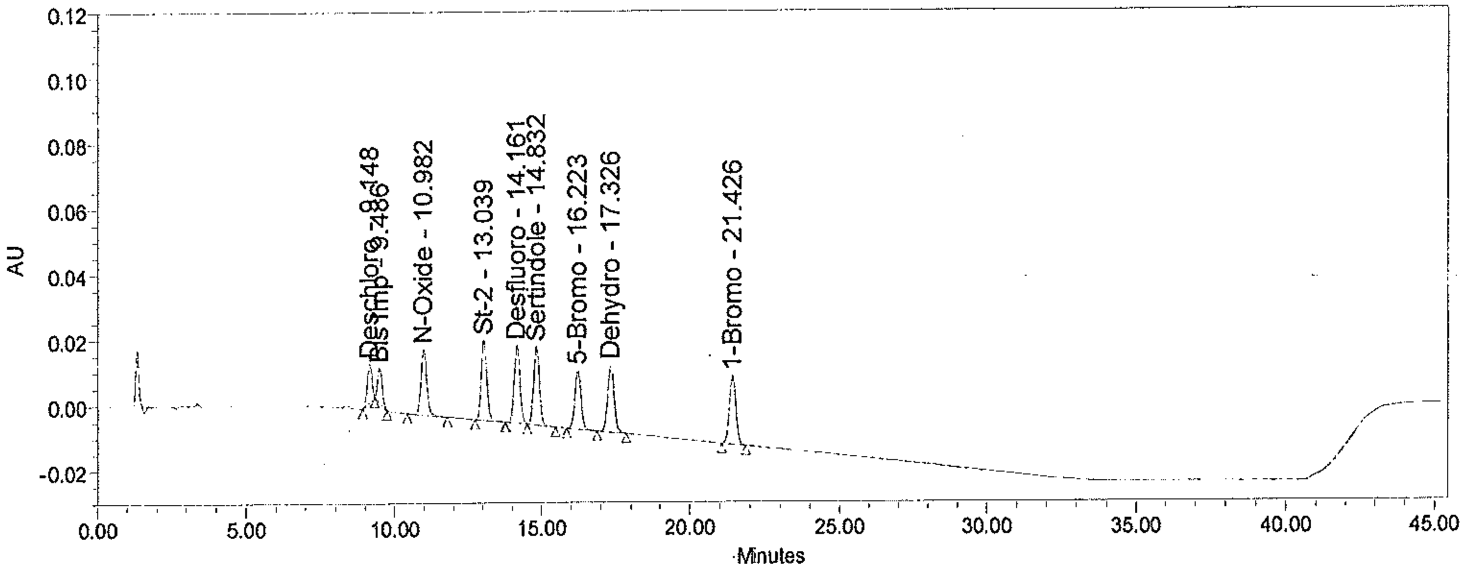

Peak Results

	Name	RT	Area (µV*sec)	% Area	RT Ratio
1	Deschloro	9.15	126531.33	5.32	0.62
2	Bis Imp	9.49	118654.03	4.99	0.64
3	N-Oxide	10.98	279757.64	11.77	0.74
4	St-2	13.04	324819.57	13.66	0.88
5	Desfluoro	14.16	327803.19	13.79	0.95
6	Sertindole	14.83	329496.88	13.86	1.00
7	5-Bromo	16.22	255223.59	10.73	1.09
8	Dehydro	17.33	304297.36	12.80	1.17
9	1-Bromo	21.43	310908.94	13.08	1.44

aptuit laurus

Analytical Development

SAMPLE INFORMATION

Sample Name:	0080/st 4/1974/130	Instrument Method Id 5847	
Sample Type:	Unknown	Acquired By:	Analyst
Vial:	34	Date Acquired:	5/12/2009 3:07:37 PM IST
Injection #:	1	Date Processed:	5/13/2009 10:49:32 AM IST
Injection Volume:	20.00 ul	Processing Method:	0080_Stage4_RS
Sample Set Id 6145		Processing Method Id 6318	
Sample Set Name:	120509_01	Proc. Chnl. Descr.:	VWD AU On nm
Project Name: Sertindole_FD_April_09		System Name LL_AD_LC_SYS012	
Result Id 6345	Result Set Id	Software: Empower 2 Software Build 2154	

Auto-Scaled Chromatogram

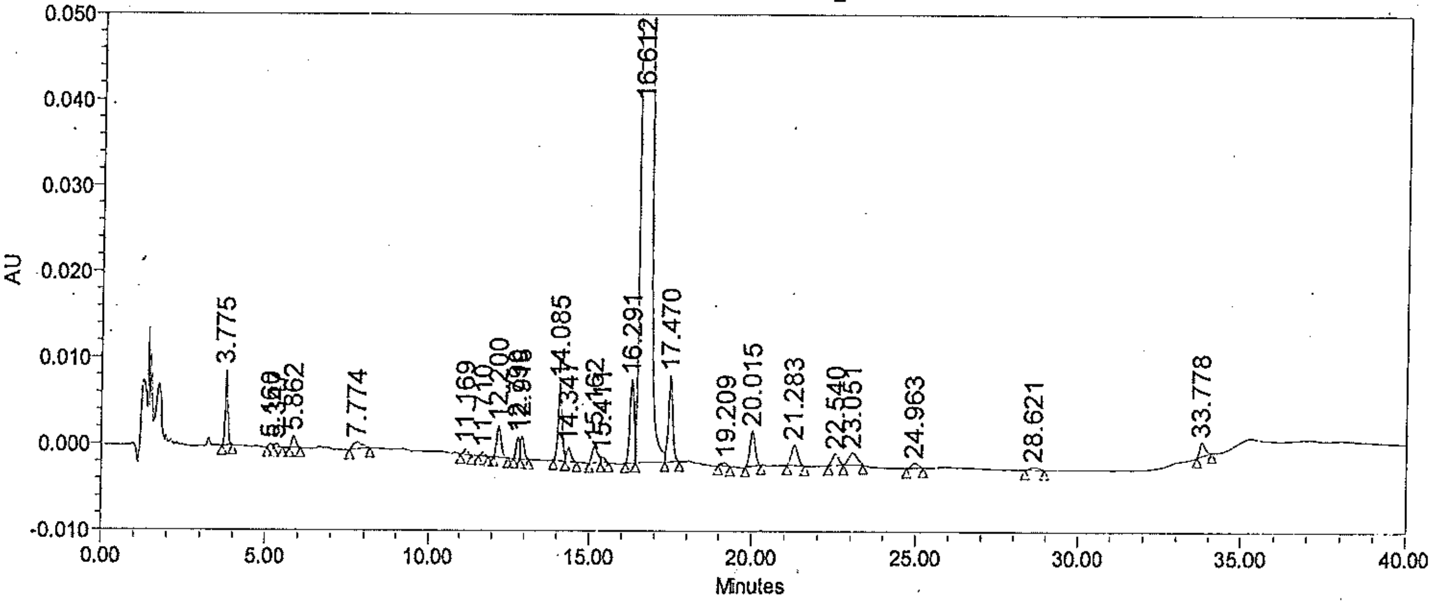

Peak Results

	Name	RT	Area	% Area	RT Ratio	USP Resolution	USP Tailing	USP Plate Count
1	Peak1	3.77	51020	0.20	0.227		1.15	9954
	Peak2	5.16	2835	0.01	0.311			
3	Peak3	5.35	3486	0.01	0.322			5138
4	Peak4	5.86	11636	0.04	0.353		1.11	9400
5	Peak5	7.77	14786	0.06	0.468	4.48	1.25	5562
6	Peak6	11.17	5532	0.02	0.672	8.05	0.98	51195
7	Peak7	11.71	3589	0.01	0.705	2.31	1.02	60372
8	Peak8	12.20	34378	0.13	0.734	2.02	1.25	37223
9	Peak9	12.79	18673	0.07	0.770			
10	Peak10	12.92	20651	0.08	0.778			
11	Peak11	14.09	77792	0.30	0.848			62500
12	Peak12	14.35	15043	0.06	0.864	0.90		47349
13	Peak13	15.16	24259	0.09	0.913	2.27		31865
14	Peak14	15.41	5877	0.02	0.928			
15	Peak15	16.29	81829	0.32	0.981			70687

SampleName 0080/st 4/1974/130

Date Acquired 5/12/2009 3:07:37 PM IST

Signature / Date: 13/05/09

Peak Results

	Name	RT	Area	% Area	RT Ratio	USP Resolution	USP Tailing	USP Plate Count
16	Stage-4	16.61	25307712	97.59	1.000	1.30	1.13	80645
17	Peak17	17.47	97279	0.38	1.052	3.47		77537
18	Peak18	19.21	4581	0.02	1.156	5.96	0.75	65531
19	Peak19	20.02	45778	0.18	1.205	2.56	1.03	70197
20	Peak20	21.28	32395	0.12	1.281	3.73	1.12	53336
21	Peak21	22.54	17330	0.07	1.357	3.35	0.98	59651
22	Peak22	23.05	24793	0.10	1.388	1.17	1.08	34474
23	Peak23	24.96	8064	0.03	1.503	4.28	1.00	49134
24	Peak24	28.62	5340	0.02	1.723	8.17	1.11	44386
25	Peak25	33.78	18313	0.07	2.033	12.45	1.32	171446

LC-MS of Sertindole

LCMS_Nov_10_039 168 (7.866) Cm (168:169-(146:159+179:194))

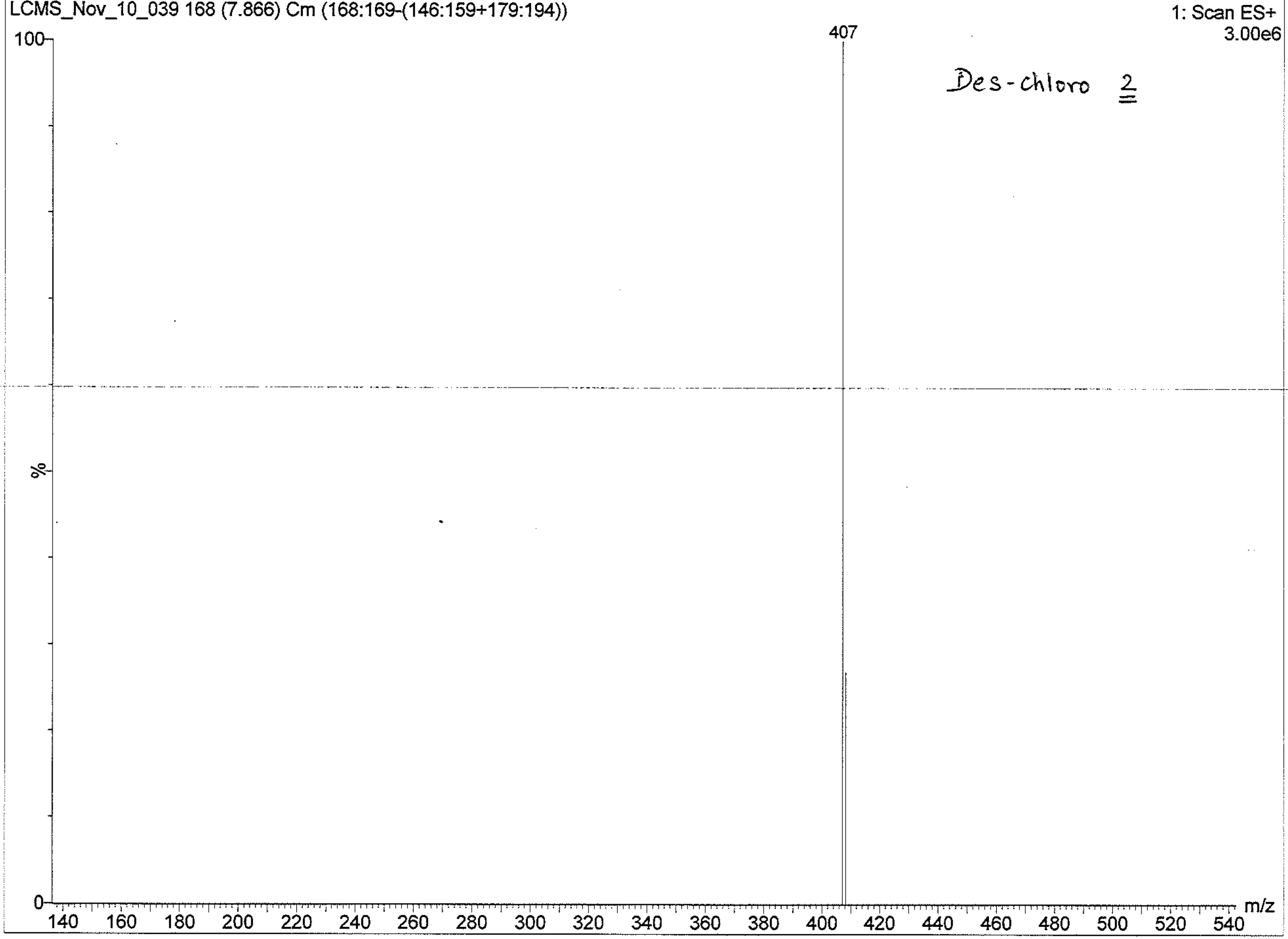

LC-MS of Sertindole

LCMS_Nov_10_039 165 (7.725) Cm (165-(125:154+175:199))

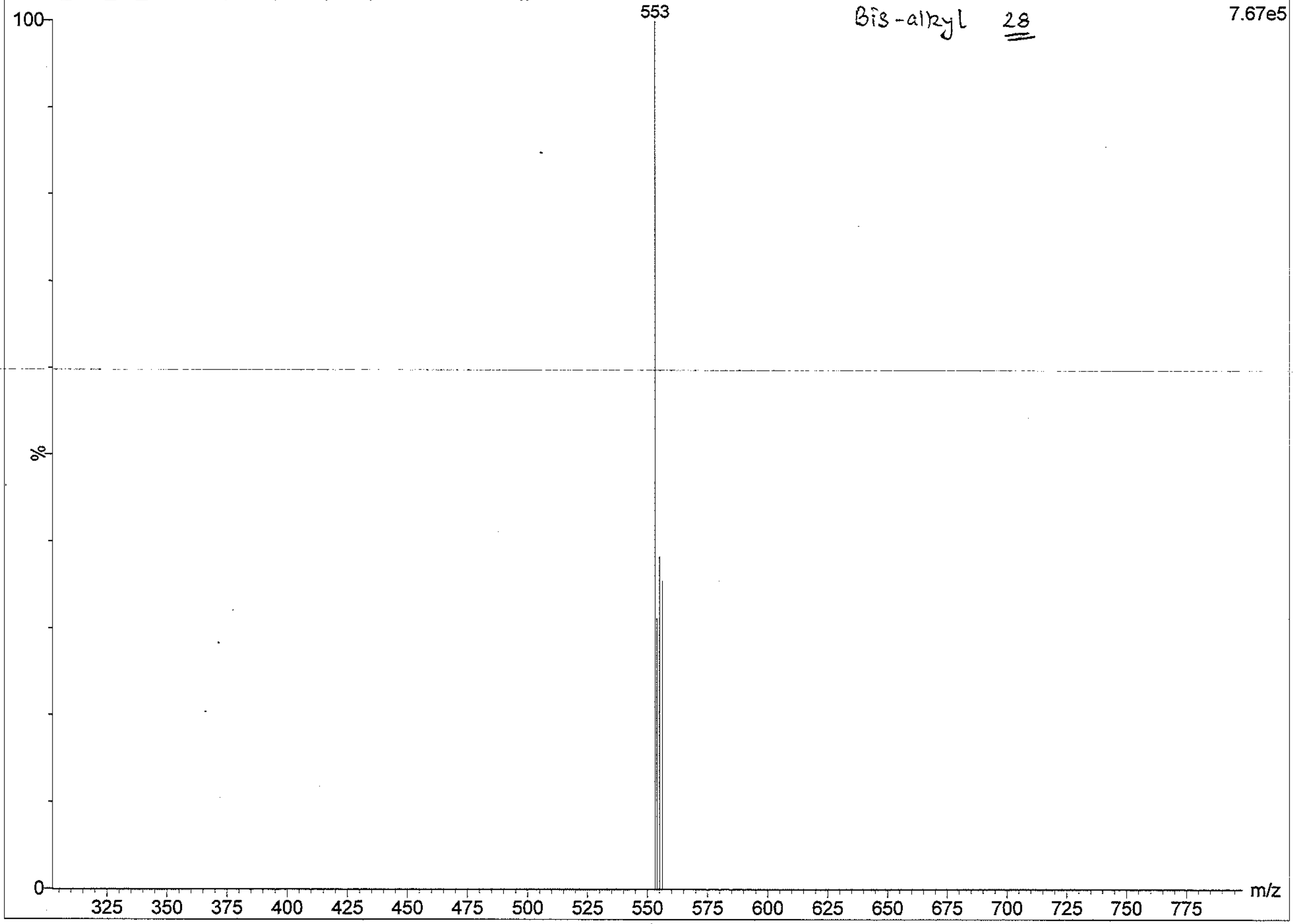

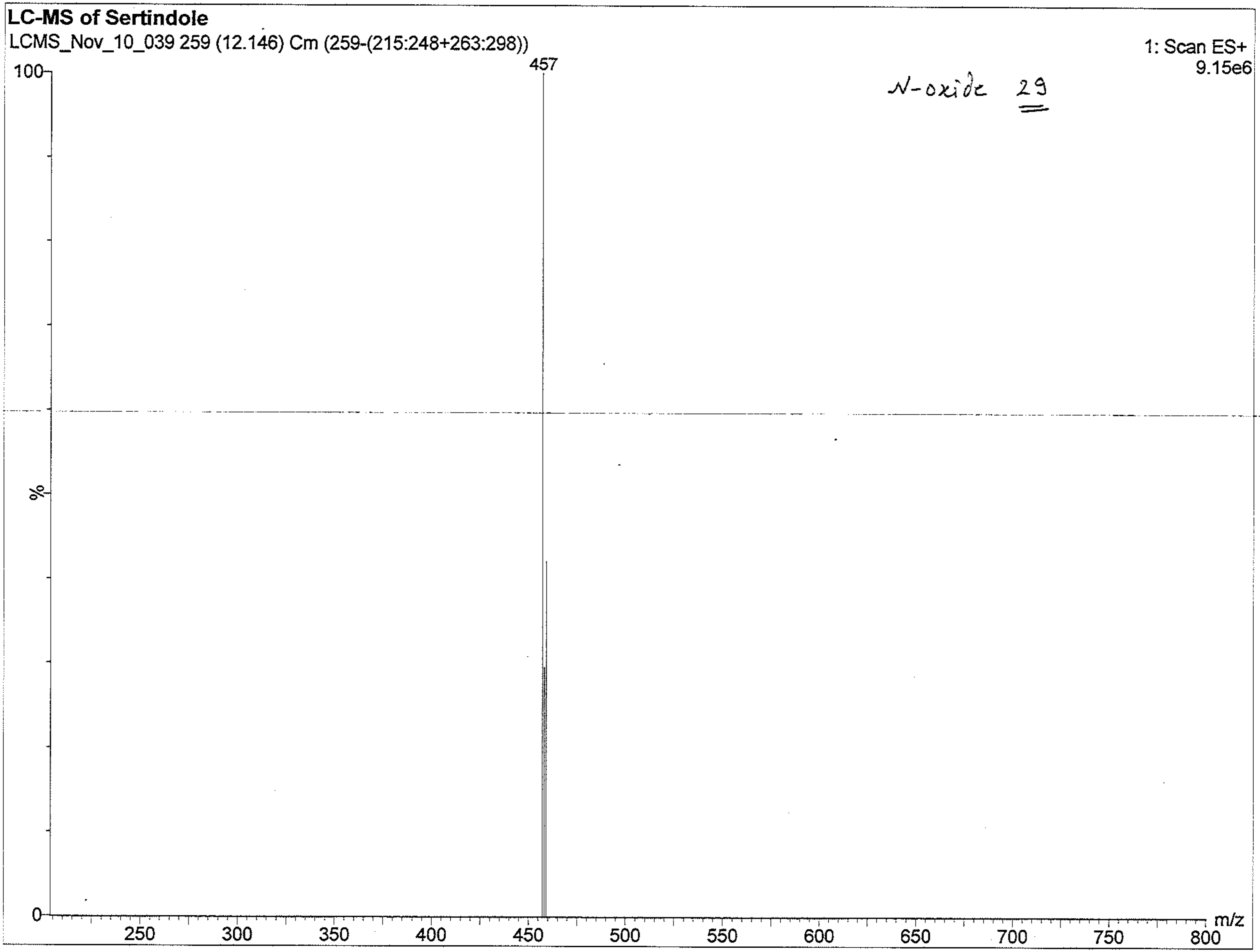
LC-MS of Sertindole
LCMS_Nov_10_039 259 (12.146) Cm (259-(215:248+263:298))
1: Scan ES+
9.15e6
N-oxide 29
457
100
%
0
250
300
350
400
450
500
550
600
650
700
750
800
m/z

LC-MS of Sertindole

LCMS_Nov_10_039 248 (11.629)

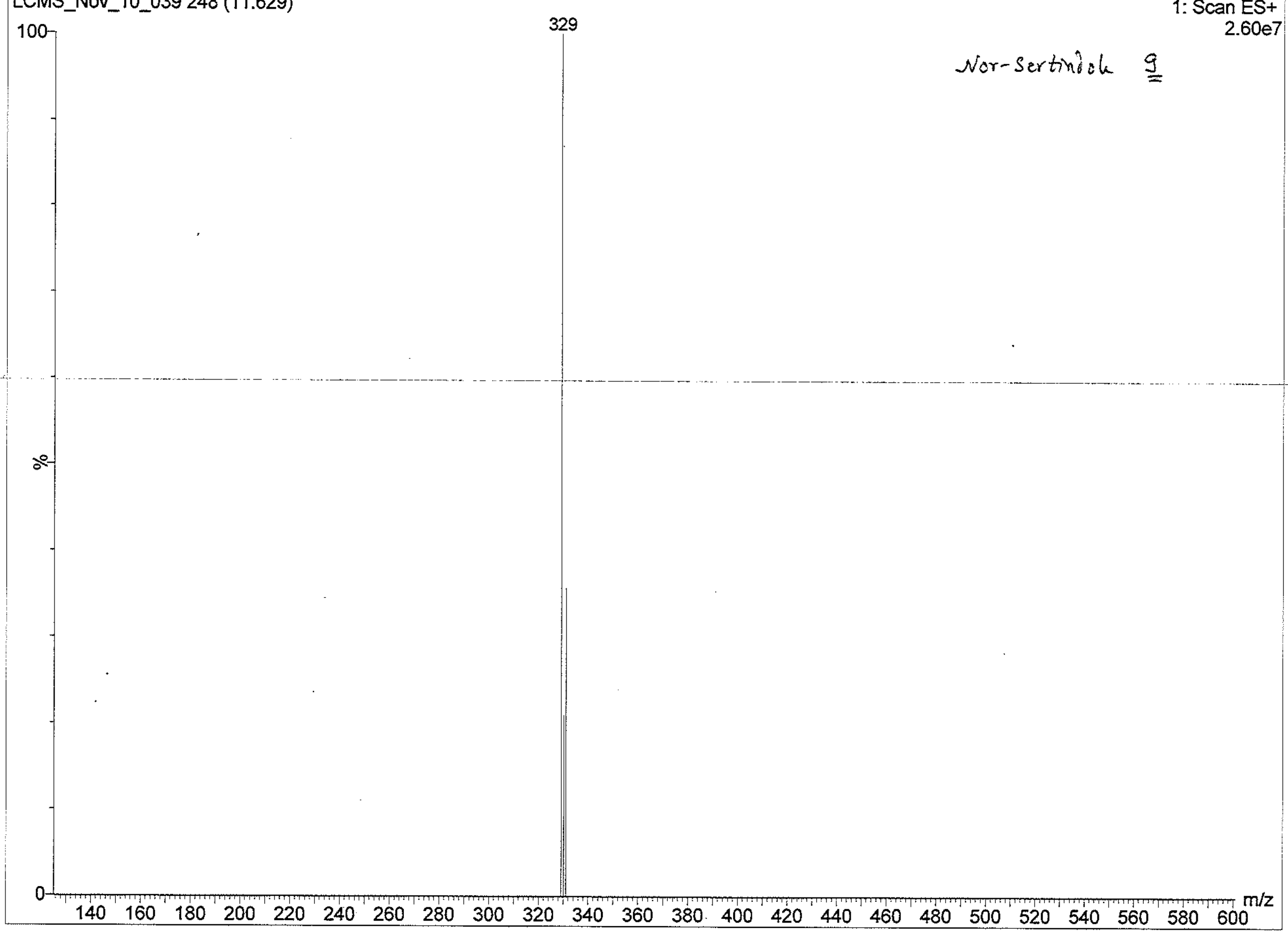

LC-MS of Sertindole

LCMS_Nov_10_039 291 (13.651) Cm (291-(270:289+295:304))

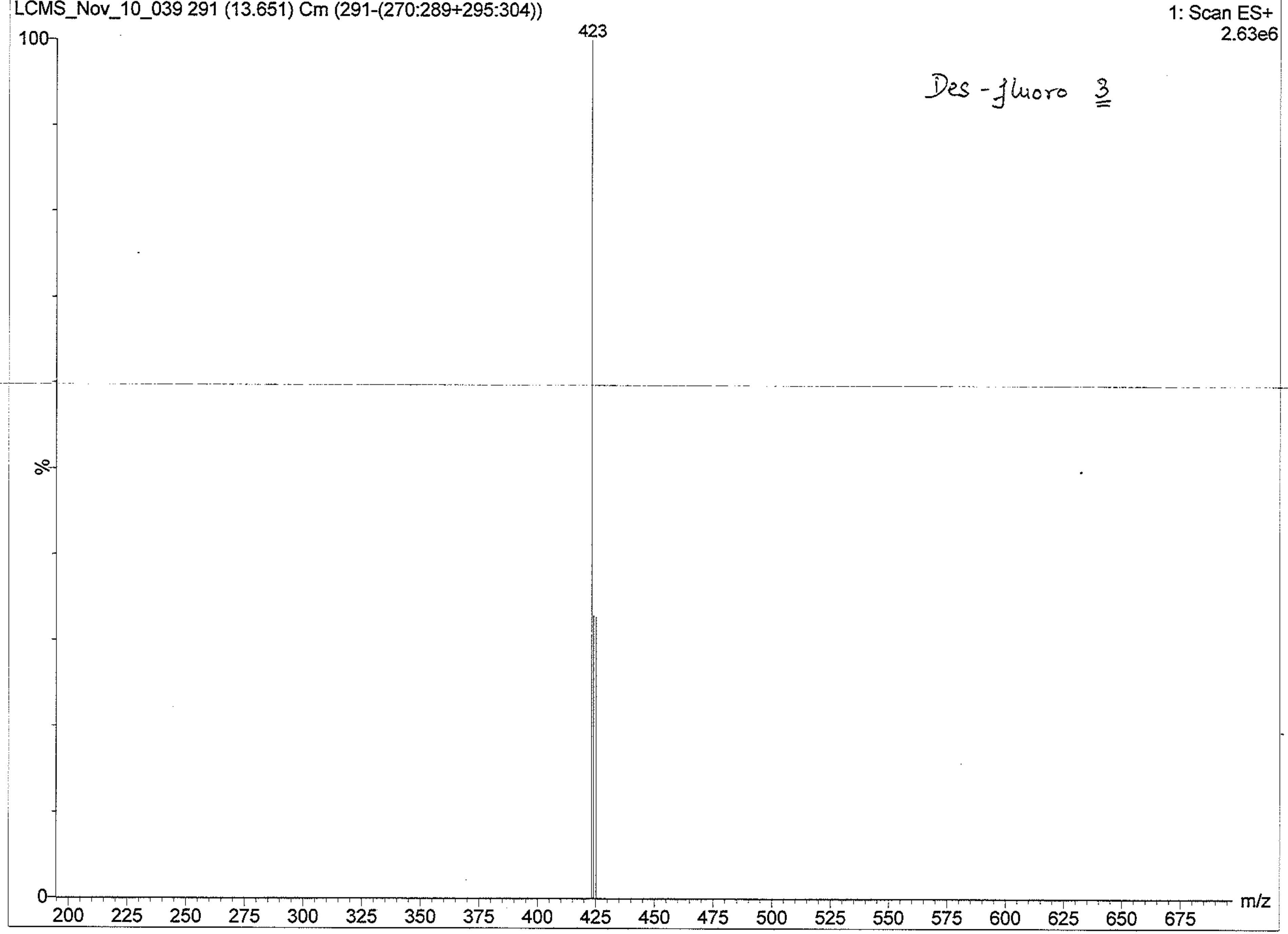

LC-MS of Sertindole

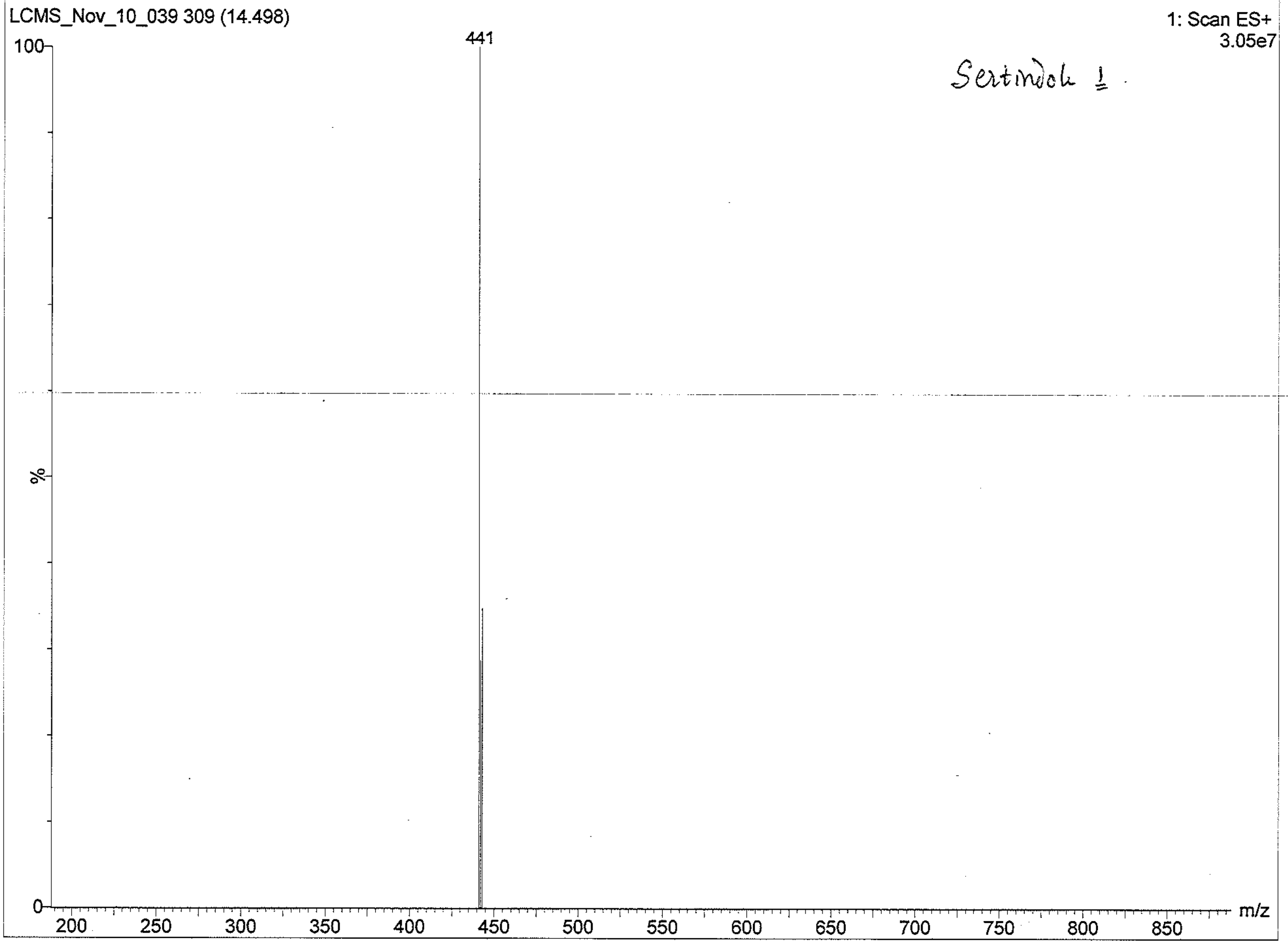

LC-MS of Sertindole

LCMS_Nov_10_039 355 (16.662)

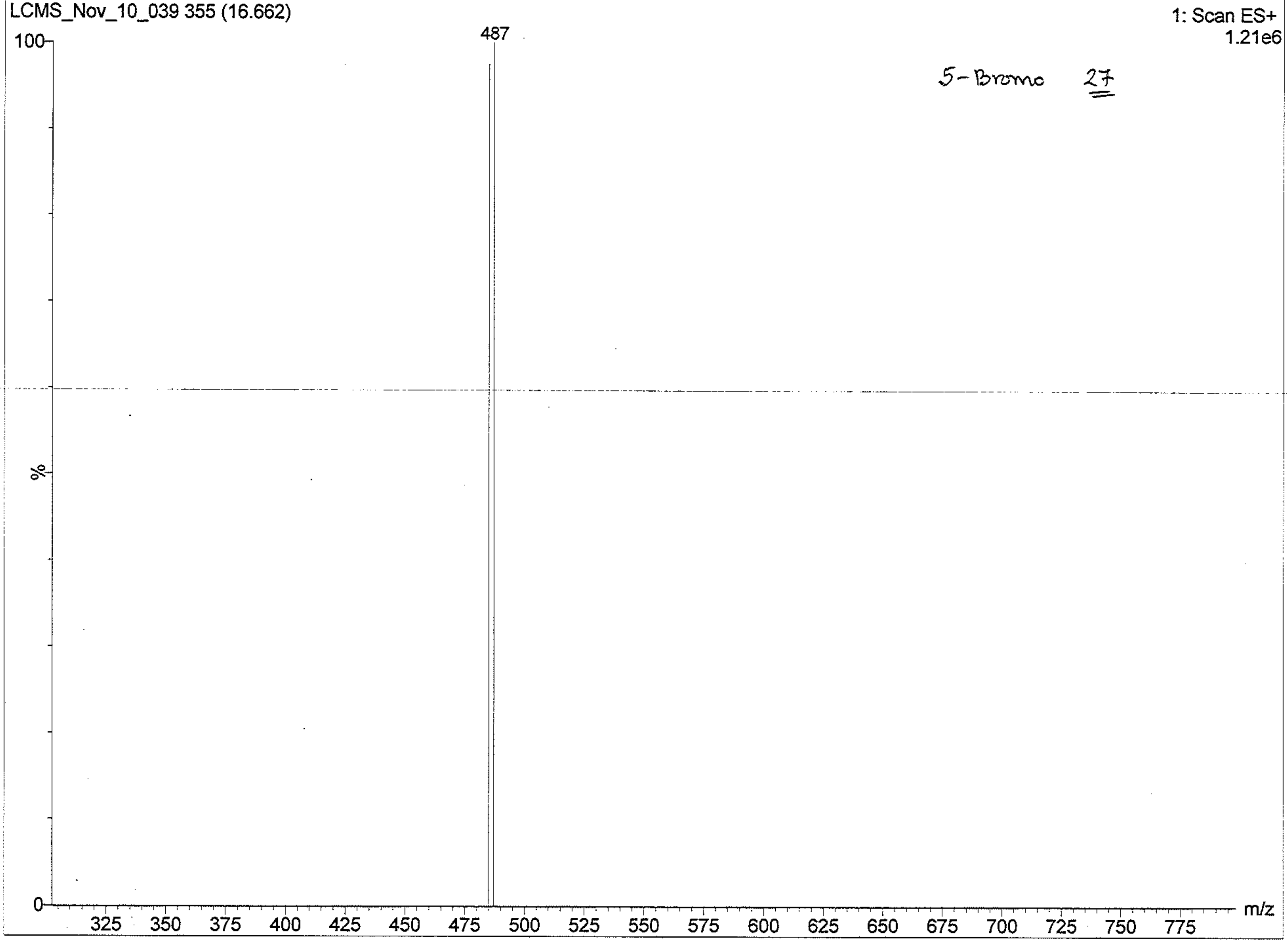

LC-MS of Sertindole

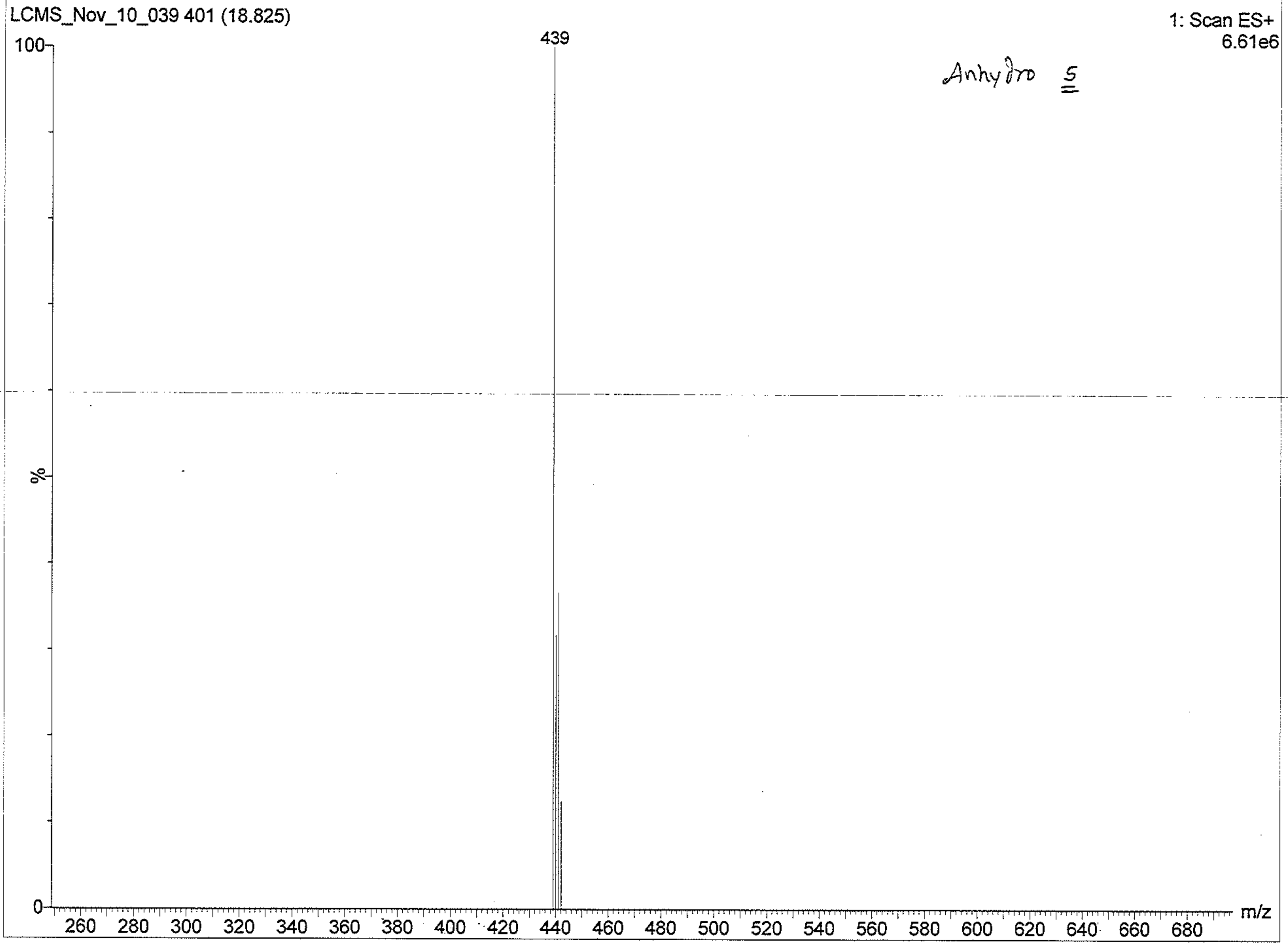

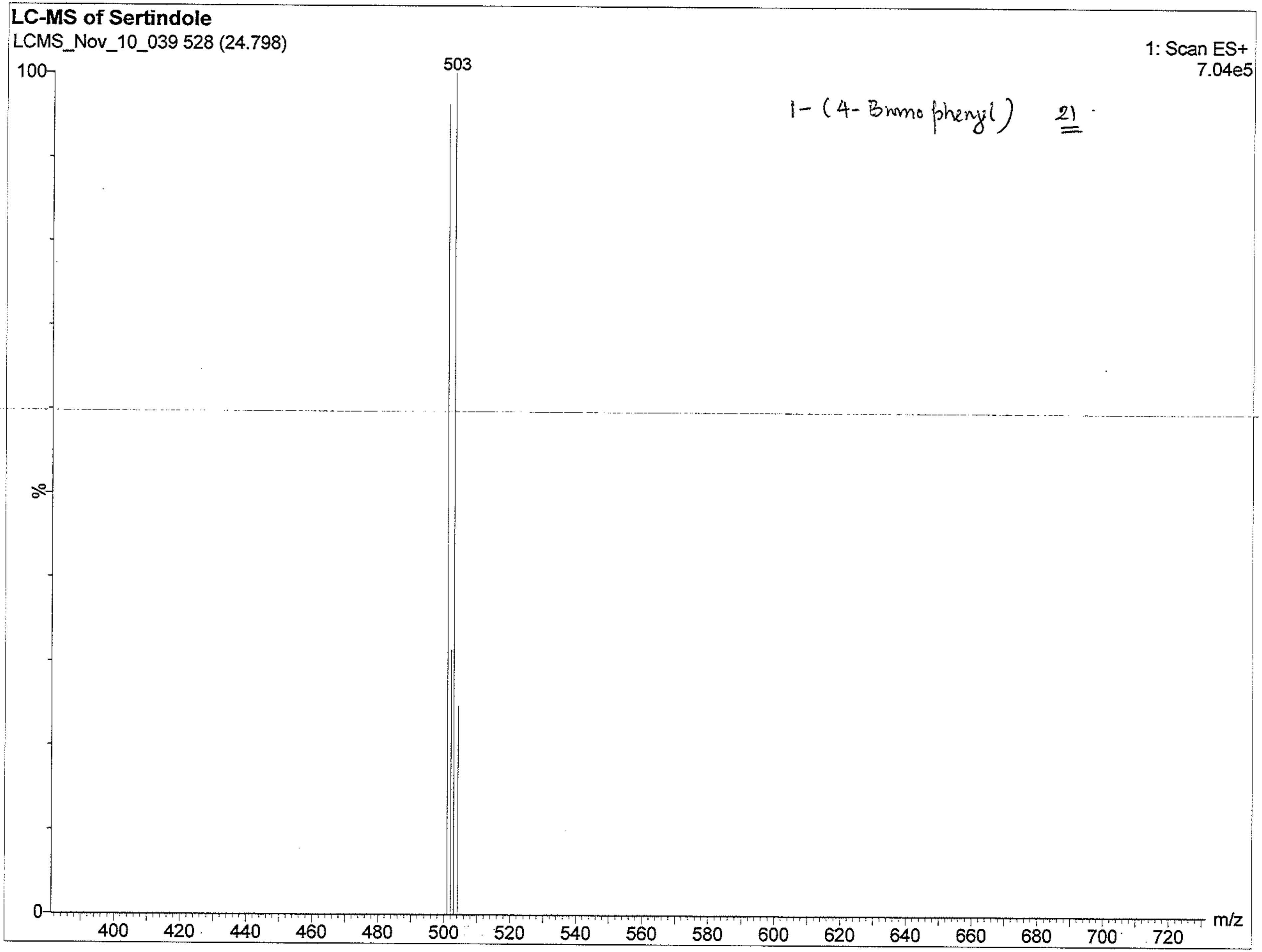
LC-MS of Sertindole
LCMS_Nov_10_039 528 (24.798)
1: Scan ES+
7.04e5
1- (4- Bromo phenyl) 21
503
100
%
0
400
420
440
460
480
500
520
540
560
580
600
620
640
660
680
700
720
m/z
